# Comparative analysis of rodent and small mammal viromes to better understand the wildlife origin of emerging infectious diseases

**DOI:** 10.1186/s40168-018-0554-9

**Published:** 2018-10-03

**Authors:** Zhiqiang Wu, Liang Lu, Jiang Du, Li Yang, Xianwen Ren, Bo Liu, Jinyong Jiang, Jian Yang, Jie Dong, Lilian Sun, Yafang Zhu, Yuhui Li, Dandan Zheng, Chi Zhang, Haoxiang Su, Yuting Zheng, Hongning Zhou, Guangjian Zhu, Hongying Li, Aleksei Chmura, Fan Yang, Peter Daszak, Jianwei Wang, Qiyong Liu, Qi Jin

**Affiliations:** 1 MOH Key Laboratory of Systems Biology of Pathogens, Institute of Pathogen Biology, Chinese Academy of Medical Sciences & Peking Union Medical College, Beijing, People’s Republic of China; 2 Collaborative Innovation Center for Diagnosis and Treatment of Infectious Diseases, Hangzhou, People’s Republic of China; 3 State Key Laboratory for Infectious Diseases Prevention and Control, National Institute for Communicable Disease Control and Prevention, Chinese Center for Disease Control and Prevention, Beijing, People’s Republic of China; 4 EcoHealth Alliance, New York, NY USA; 5 Yunnan Institute of Parasitic Diseases, Puer, People’s Republic of China

**Keywords:** Rodents, Small mammals, Virome, Viral evolution, Emerging infectious diseases

## Abstract

**Background:**

Rodents represent around 43% of all mammalian species, are widely distributed, and are the natural reservoirs of a diverse group of zoonotic viruses, including hantaviruses, Lassa viruses, and tick-borne encephalitis viruses. Thus, analyzing the viral diversity harbored by rodents could assist efforts to predict and reduce the risk of future emergence of zoonotic viral diseases.

**Results:**

We used next-generation sequencing metagenomic analysis to survey for a range of mammalian viral families in rodents and other small animals of the orders *Rodentia*, *Lagomorpha*, and *Soricomorpha* in China. We sampled 3,055 small animals from 20 provinces and then outlined the spectra of mammalian viruses within these individuals and the basic ecological and genetic characteristics of novel rodent and shrew viruses among the viral spectra. Further analysis revealed that host taxonomy plays a primary role and geographical location plays a secondary role in determining viral diversity. Many viruses were reported for the first time with distinct evolutionary lineages, and viruses related to known human or animal pathogens were identified. Phylogram comparison between viruses and hosts indicated that host shifts commonly happened in many different species during viral evolutionary history.

**Conclusions:**

These results expand our understanding of the viromes of rodents and insectivores in China and suggest that there is high diversity of viruses awaiting discovery in these species in Asia. These findings, combined with our previous bat virome data, greatly increase our knowledge of the viral community in wildlife in a densely populated country in an emerging disease hotspot.

**Electronic supplementary material:**

The online version of this article (10.1186/s40168-018-0554-9) contains supplementary material, which is available to authorized users.

## Background

Approximately two thirds of emerging infectious diseases (EIDs) that affect humans originate from bats, rodents, birds, and other wildlife [1–3]. In many of these reservoir host species, emerging viruses appear to be well adapted, with little or no evidence of clinical disease. However, when these viruses spill over into humans, the effects can sometimes be devastating [4–6]. Previously, our limited knowledge of the viral population and ecological diversity harbored by wildlife have complicated the study of EIDs. Thus, comprehensive understanding of the viral community present in wildlife, as well as the prevalence, genetic diversity, and geographical distribution of these viruses, could be valuable for prevention and control of wildlife-origin EIDs [7].

The order *Rodentia* is the largest mammalian order, with 33 families and 2,277 species (~ 43% of all mammal species). They live in close contact with humans and their domestic animals and act as a bond between humans, domestic animals, arthropod vectors (ticks, mites, fleas), and other wildlife [8–10]. This interface with humans has led to the rodent origin of important zoonotic viruses including members of the family *Arenaviridae*, *Hantaviridae*, *Reoviridae*, *Togaviridae*, *Picornaviridae*, and *Flaviviridae* [11–18]. Many of these viruses cause severe disease in humans (e.g., Lassa virus; tick-borne encephalitis virus, TBEV; lymphocytic choriomeningitis virus, LCMV; Sin Nombre virus; Hantaan virus, HTNV; Seoul virus, SEOV; and Puumala virus); have only recently been discovered (e.g., Whitewater Arroyo virus and Lujo virus); or appear to have a wider geographical range than originally thought (e.g., Junin virus, Guanarito virus, Machupo virus, and Sabia virus), suggesting that further viral discovery studies in wild rodent populations may be valuable for public health [8, 11–13, 15, 19–25]. Recent reports of rodent viruses have enabled new hypotheses regarding the evolution of hepaciviruses and the origin of coronaviruses (CoVs) and picornaviruses (PicoVs) such as hepatitis A virus [26–29].

China is a megadiversity country and harbors ~ 200 rodent species from 12 families [30]. To develop baseline data on the origin of existing viral EIDs and identify other potential zoonotic viral reservoir hosts, we have conducted a series of viral surveys from rodents, bats, and other small animals and have simultaneously constructed online viral databases of these animals (DBatVir and DRodVir, http://www.mgc.ac.cn/) since 2010 [31–34]. In the current study, 3,055 small mammal individuals of 55 species from the orders *Rodentia*, *Lagomorpha*, and *Soricomorpha* across China were sampled by pharyngeal and anal swabbing. Virome analysis was then conducted to outline the viral spectrum within these samples. On the basis of virome data, we describe the community, genetics, evolution, and ecological distribution characteristics of viruses and determined whether these features change with their host species and locations. The identification of novel mammal viruses provides new clues in the search for the origin or evolution pattern of human or animal pathogens such as hantaviruses (HVs), arenavirus (AreVs), CoVs, and arteriviruses (ArteVs).

## Results

### Animal sampling

**Fig. 1:**
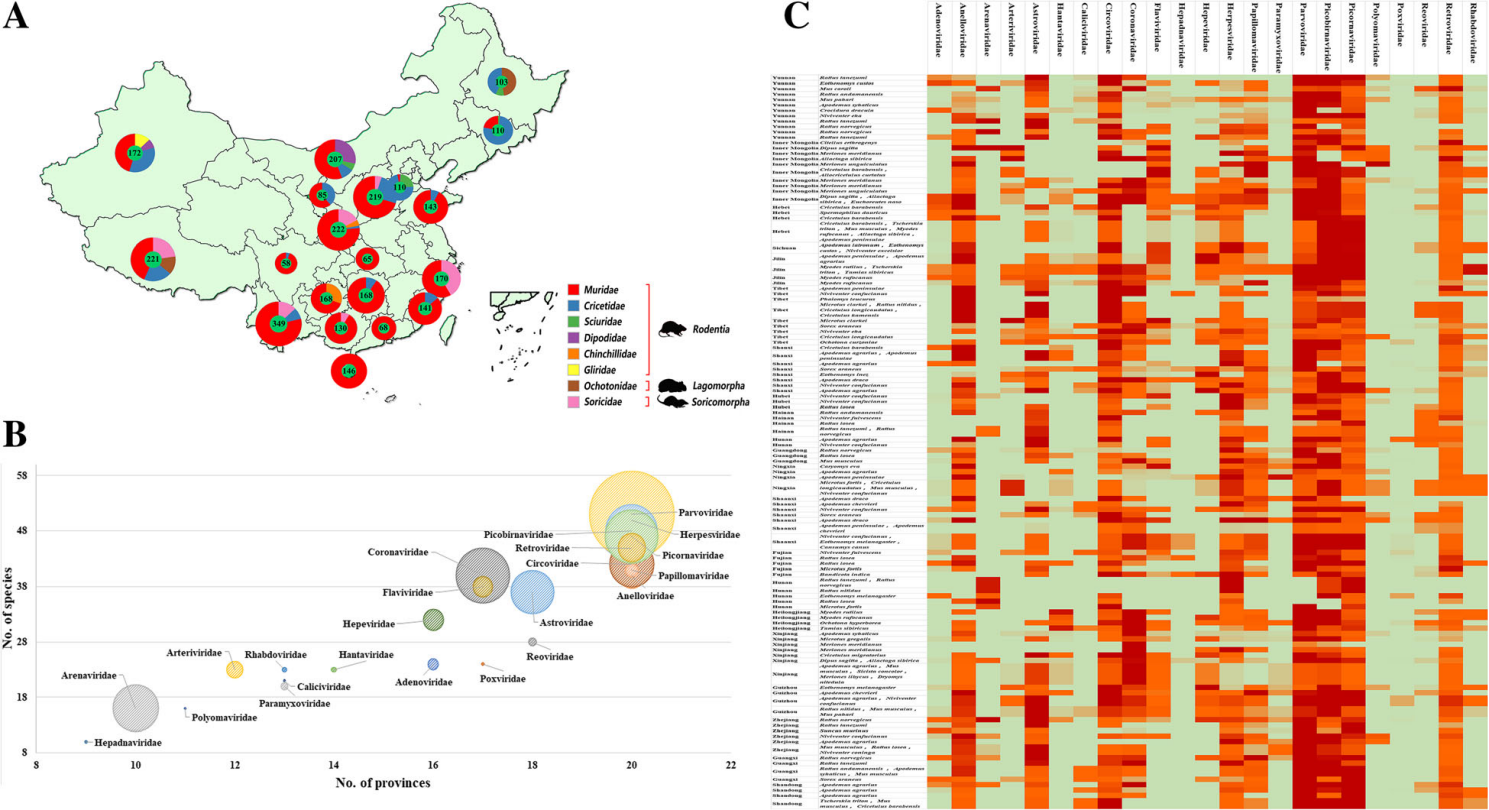
**a** Numbers of animal samples from various provinces. The numbers of the 3,055 samples belonging to the 55 species of eight families identified are indicated by a pie chart for each province. The numbers of samples from the 55 species and the provinces and dates of collection are detailed in Additional file 1: Table S1. **b** The prevalence diagram of each viral family related to province, animal species, and reads number. The *X* axis represents how many provinces certain viral family presents; the *Y* axis represents how many animal species certain viral family presents; and the sizes of these circles represent the sizes of reads numbers of viral families. **c** Heatmap based on the normalized sequence reads of 23 families of mammalian viruses in each pooled sample. The species are listed in the right text column. Location information is provided in the life text column. The names of the mammalian viral families are presented in the top text row. The boxes colored from green to red represent the viral reads, which were normalized by average viral genome size and total sequencing reads in each pool

Pharyngeal and anal swabs were collected from 3,055 individual small mammals captured from July 2013 to July 2016 in 20 provinces across China (Fig. 1a and Additional file 1: Table S1). These comprised 50 rodent species of the families *Muridae*, *Cricetidae*, *Sciuridae*, *Dipodidae*, *Chinchillidae*, and *Gliridae*; two lagomorphs of the family *Ochotonidae*; and three soricomorphs of the family *Soricidae* that reside in urban, rural, and wild areas throughout China. The most common species sampled were *Apodemus agrarius*, *Niviventer confucianus*, *Rattus norvegicus*, *Rattus tanezumi*, *Rattus losea*, and *Sorex araneus*. Due to repeated sampling of some species in the same location, swabs were combined into 110 pools for analysis.

### Metagenomic analysis and virome overview

A total of 65.6 GB of nucleotide data (693,985,331 valid reads, 100 bp in length) was obtained. Reads classified as eukaryotes or prokaryotes and those with no significant similarity to any amino acid (aa) sequence in the NR database were discarded, leading to 12,073,729 reads best matched with viral protein sequences in the NR database (~ 1.7% of the total sequence reads). The number of virus-associated reads in each lane varied between 2,774 and 658,417.

A wide range of DNA and RNA virus groups were covered by these reads. Virus-associated reads were assigned into 70 families of double-stranded (ds)DNA viruses, dsRNA viruses, retro-transcribing viruses, single-stranded (ss)DNA viruses, and ssRNA viruses in the virus root (Additional file 1:Table S2). Dietary habits and other host traits were used to exclude insect viruses, fungal viruses, plant viruses, and phages as described previously [31]. The remaining 7,148,634 sequence reads (~ 59.2% of the total viral hits) were assigned into 23 families of mammalian viruses (Additional file 1: Tables S3 and Additional file 2: Table S14). The prevalence diagram of each viral family related to province, animal species, and reads number was shown in Fig. 1b. The relative abundances of the 23 viral families in pooled samples of different provinces and animals were calculated by normalizing sequence reads and were shown in Fig. 1c. Viral reads from the families *Herpesviridae*, *Picobirnaviridae*, *Anelloviridae*, *Circoviridae*, *Retroviridae*, *Astroviridae*, *Coronaviridae*, and *Picornaviridae*, and the subfamily *Parvovirinae*, were widely distributed in different animal species from different regions of China. Viral reads from the families *Adenoviridae*, *Poxviridae*, *Papillomaviridae*, *Reoviridae*, *Arenaviridae*, *Arteriviridae*, *Hantaviridae*, *Caliciviridae*, *Flaviviridae*, *Hepeviridae*, *Rhabdoviridae*, and *Paramyxoviridae* were found in fewer species. Many of the sequence reads related to mammalian viruses showed low nucleotide (nt) and aa sequence identity with known viruses.

The abundance of virus strains in these mammalian viral families was further confirmed on a sample-by-sample basis by PCR screening. In total, 586 positive results were obtained, and 203 viruses from representative positive samples (representative virus strains represent viruses with identical or almost identical sequences (≥ 97% nt identity) from the same host species at the same location) were selected for genomic or partial genomic sequencing as quasi-species of these viruses (Additional file 1: Table S4). According to the genus and species demarcation criteria in each viral family established by the International Committee on Taxonomy of Viruses (ICTV; https://talk.ictvonline.org/), these viruses may represent 160 new species and at least seven new genera. Although sequence reads apparently from the families *Hepadnaviridae* and *Poxviridae* were occasionally present in some of the samples, it was not possible to amplify viral sequences from these samples, likely due to low viral loads. No virus was detected from samples of lagomorphs, this finding may reveal that the virome of lagomorphs is far less abundant than that of rodents and soricomorphs.

### Ecological characteristics of identified viruses

**Fig. 2:**
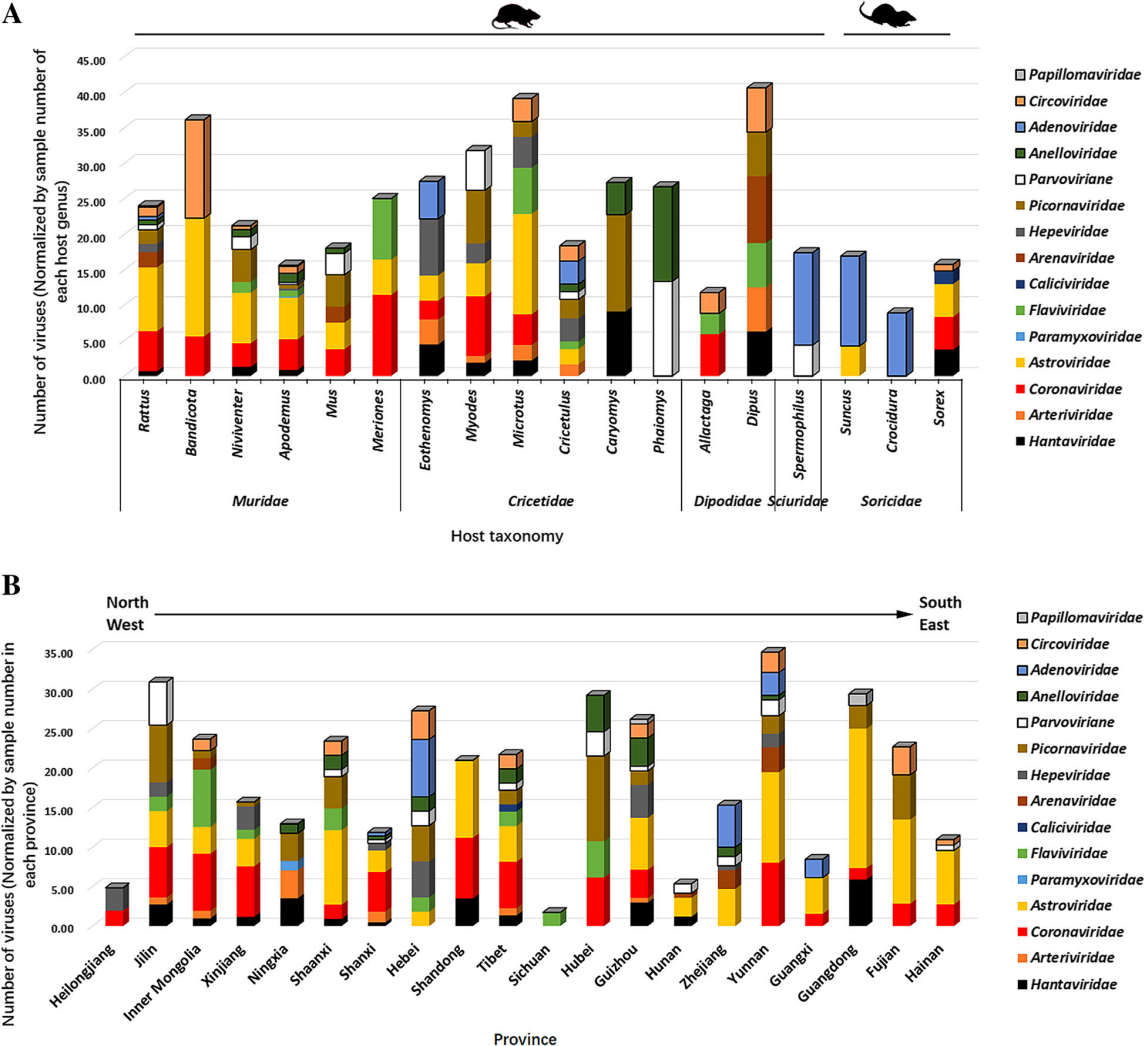
**a** Overview of the diversity and abundance of the identified RNA and DNA viruses classified by viral family and host genus. **b** Overview of the diversity and abundance of the identified RNA and DNA viruses classified by viral family and geographical distribution. The number of viruses obtained by sample-by-sample PCR screening was normalized by sample size of each host genus (**a**) or province (**b**)

By classifying all positive results into each viral family and host genus and then normalizing the virus number according to the sampling number of each host genus, our analysis revealed significant differences among hosts in terms of virus composition and abundance (Fig. 2a). Animals of the families *Muridae* and *Cricetidae* acted as major reservoirs for diverse mammalian viruses in China. Although HVs were detected in animals of the families *Muridae*, *Cricetidae*, *Dipodidae*, and *Soricidae*, most of them were of low abundance when compared with other RNA viruses. AreVs were only detected in animals of the families *Muridae* and *Dipodidae* with low abundance, and ArteVs were only detected in animals of the families *Cricetidae* and *Dipodidae* with low abundance. Animals of the family *Muridae* and the subfamily *Arvicolinae* were the main hosts of CoVs and astroviruses (AstVs) with high viral richness. Notably, although limited samples from animals of the family *Dipodidae* were collected (73 animals from four species) for virome analysis, *Allactaga* and *Dipus* still harbored diverse viruses with high abundance.

To further describe the viral diversity and abundance differences among geographical locations, the virus number of each family was assigned and normalized by the sample size in each province (Fig. 2b). Since many host rodent species such as *Rattus* and *Apodemus* species are broadly distributed throughout China, most RNA and DNA viruses with high (e.g., CoVs, AstVs, and PicoVs) or low (e.g., HVs and parvoviruses (ParVs)) richness were detected in diverse locations throughout China and showed no obvious geographical preference (except some regions that harbored unusual host species showed geographical restriction of certain viruses). However, ArteVs and flaviviruses tended to be frequently detected in provinces in the north, west, and middle of China with low or medium abundance, and such viruses were not detected in southern and coastal areas. In contrast, except *Dipus*-related AreVs detected in Inner Mongolia and showed distinct genome sequence, all other Old World AreVs were only present in three southern areas, Yunnan, Hunan, and Zhejiang.

### Evolutionary characteristics of RNA viruses

#### HVs

**Fig. 3:**
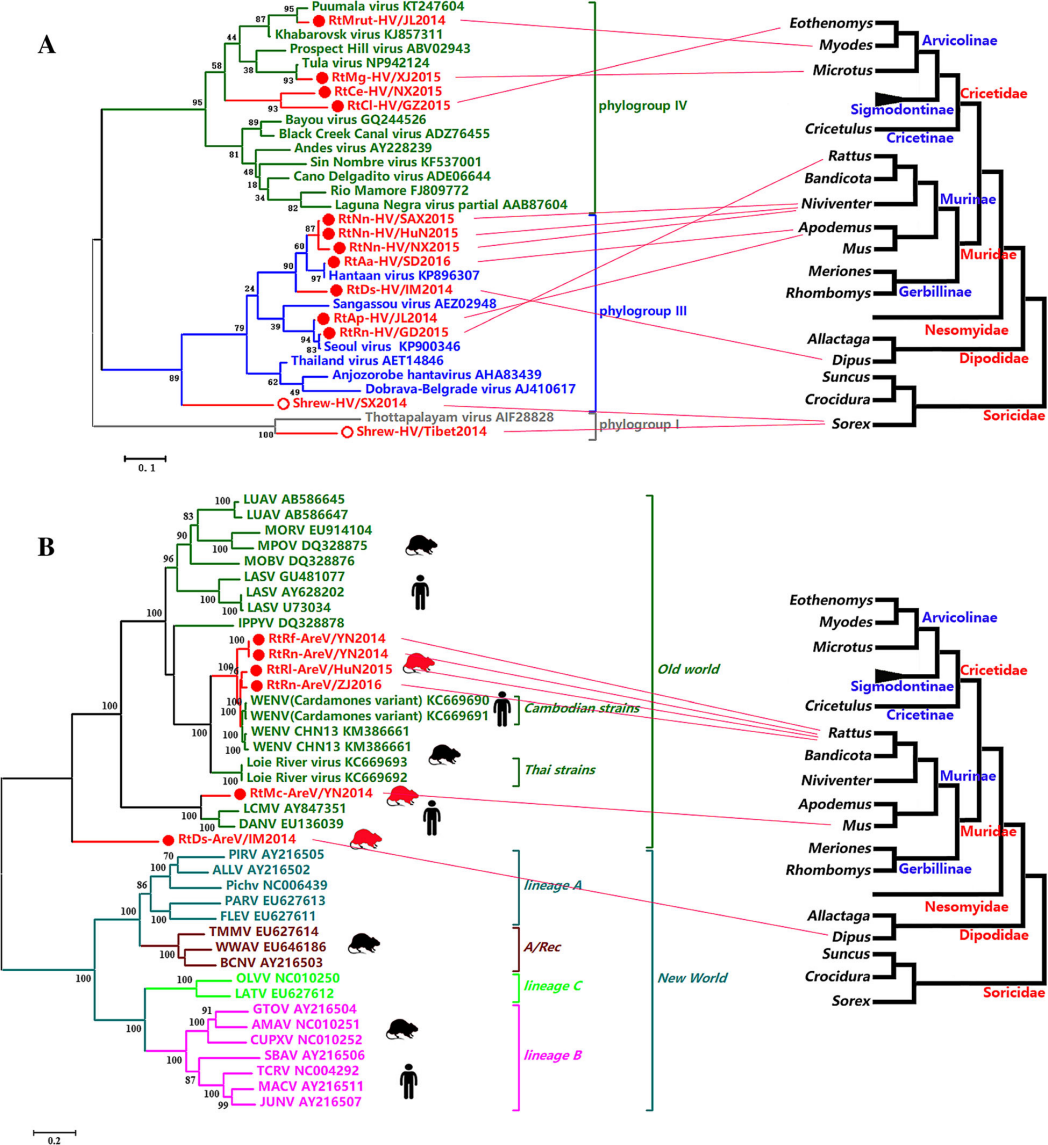
**a** Phylogenetic tree based on the partial L protein sequences of HVs. **b** Phylogenetic tree based on the complete L proteins of AreVs. The viruses found in this study are labeled in red font. The evolutionary lineages of involved hosts on the right were drawn based on mt-*cyt b* from genus to family according to previous reports [7, 9, 68–70]. The relationships between viruses and their hosts were linked by red lines

An L-segment-based, pan-HV PCR was used to sequence HVs. A total of 32 samples from 11 provinces were positive for HV (Additional file 1: Table S4). In rodents, PCR products of the expected size were amplified from *Niviventer confucianus*, *Apodemus peninsulae*, *Apodemus agrarius*, and *Rattus norvegicus* of the family *Muridae*, *Myodes rutilus*, *Microtus gregalis*, and *Caryomys eva*; *Eothenomys melanogaster* of the family *Cricetidae*; and *Dipus sagitta* of the family *Dipodidae*. In *Soricomorpha* insectivores, expected PCR products were obtained from *Sorex araneus* of the family *Soricidae*. Thirteen representative viral strains of the 32 positive results were selected for phylogenetic analysis (Fig. 3a). Eight viruses from three rodent families were assigned into phylogroup III. Four viruses clustered in the HTNV clade, and two viruses clustered in the SEOV clade, with high sequence similarities (95–99% aa identities). RtDs-HV/IM2014 identified from *Dipus sagitta* was located between HTNVs and SEOVs (85–93% aa identities). Shrew-HV/SX2014 was located outside the rodent HV clade within this phylogroup. Four viruses of cricetids were assigned to phylogroup IV and comprised Puumala virus and Tula virus (97% aa identity with known viruses), and two viruses, RtCe-HV/NX2015 and RtCl-HV/GZ2015, which formed a separate clade with < 90% aa identity with any other viruses from phylogroup IV. Another shrew HV, Shrew-HV/Tibet2014, was located in phylogroup I.

#### AreVs

Nineteen rodent samples from Zhejiang, Hunan, Yunnan, and Inner Mongolia were positive for AreVs. Six strains were selected for genome sequencing and further analyses (Additional file 1: Table S4), and all were assigned to the Old World complex (Fig. 3b, Additional file 3: Figures S1 and S2). Four viruses identified from *Rattus tanezumi*, *Rattus norvegicus*, and *Rattus losea* captured in Zhejiang, Hunan, and Yunnan clustered in the Wenzhou virus (WENV) clade with high sequence similarity (87–89% aa identities for L, 93% for G, and 95% for N, Additional file 1: Table S5). RtMc-AreV/YN2014 from *Mus caroli* was distant from other AreVs and clustered with DANV and LCMVs with short branch lengths. RtDs-AreV/IM2014 of *Dipus sagitta* appeared to represent a separate evolution being distant from all other AreVs in the Old World complex.

#### ArteVs

**Fig. 4:**
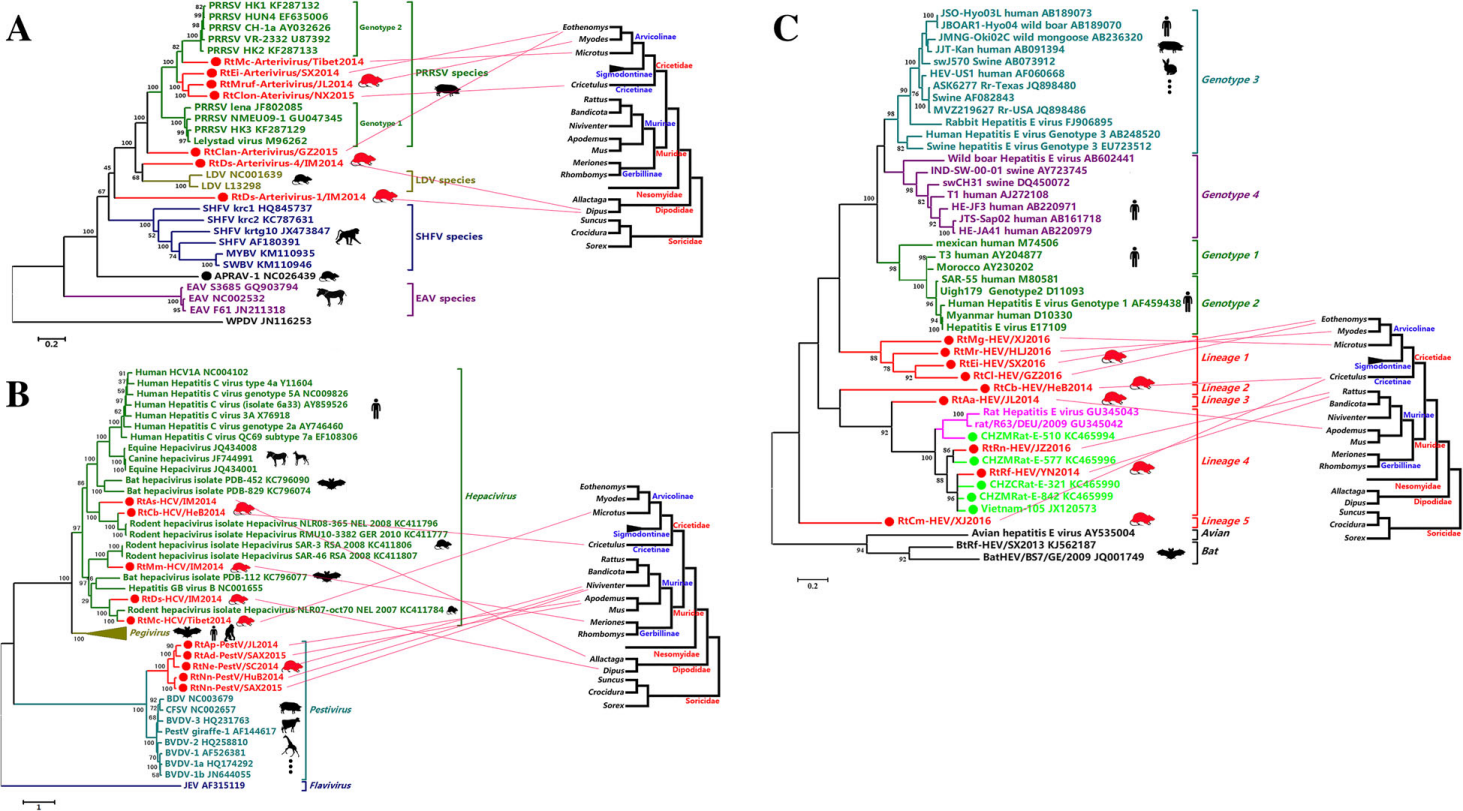
**a** Phylogenetic tree based on the complete aa sequences of ORF1b of ArteVs. **b** Phylogenetic tree based on the polyproteins of hepacivirus and PestVs. **c** Phylogenetic tree based on the complete ORF1 sequences of HEVs. The viruses found in this study are labeled in red font. The evolutionary lineages of involved hosts on the right were drawn based on mt-*cyt b* from genus to family according to previous reports [7, 9, 68–70]. The relationships between viruses and their hosts were linked by red lines

Twelve rodent samples of six species from six provinces were positive for ArteVs. Seven strains were determined for genome sequences (Additional file 1: Table S4). Five virus strains identified from *Microtus clarkei*, *Eothenomys inez*, *Eothenomys melanogaster*, *Myodes rufocanus*, and *Cricetulus longicaudatus* in five provinces appeared to be closely related to porcine reproductive and respiratory syndrome virus (PRRSV) with higher sequence similarity than those with other members of the family *Arteriviridae* (60.1–73.7% versus 25.7–54.2% aa identity for ORF1b, compared with equine arteritis virus, lactate dehydrogenase-elevating virus (LDV) of mice, simian hemorrhagic fever virus, and wobbly possum disease virus; Additional file 1: Table S6). The other two viruses detected in *Dipus sagitta* showed low sequence similarity with known ArteVs (25.2–55.9% aa identity for ORF1b, Additional file 1: Table S6). Genomic structure and phylogenetic analysis (Fig. 4a, Additional file 3: Figures S3 and S4) indicated that four of the five PRRSV-related viruses were assigned into the PRRSV species as an intermediate between genotype 1 and 2. Furthermore, these four viruses showed a closer relationship to genotype 2 than genotype 1 in the PRRSV species. RtClan-Arterivirus/GZ2015 clustered with the clade of PRRSV species but appeared to represent a separate evolutionary lineage. The other two ArteVs identified from *Dipus sagitta* in Inner Mongolia formed separate branches that were evolutionarily distant from all known members of the *Arteriviridae*.

#### *Flaviviridae*; *Hepaciviruses*, *Pestiviruses* (PestVs), and TBEV

A total of 35 rodent samples were identified as containing members of the family *Flaviviridae*. Eleven strains (five hepaciviruses, five PestVs, and one TBEV) were selected for genome sequencing (Additional file 1: Table S4). The five novel rodent hepaciviruses were assigned into different clades under the genus *Hepacivirus* with varied sequence similarity to other rodent hepaciviruses (14.1–65.6% aa identities; Fig 4b and Additional file 1: Table S7) [27]. The five PestVs showed low sequence similarity with known PestVs from artiodactylid hosts (< 40% aa identity) and formed a distinct novel rodent virus lineage distant from all other members of the genus *Pestivirus*. A TBEV, RtMg-TBEV/XJ2015 in *Microtus gregalis* from Xinjiang, showed high (> 99%) nt and aa identities with known TBEV found in ticks (*Ixodes scapularis*) in Xinjiang (Additional file 3: Figure S5).

#### Hepatitis E viruses (HEVs)

Thirty-one rodent samples were HEV positive, and the genome sequences of nine viruses were confirmed (Additional file 1: Table S4). All had < 44.5% aa homology in ORF1 compared with HEVs from other hosts (Fig. 4c and Additional file 1: Table S8). These nine viruses comprised five lineages; all of which represented novel clades of rodent HEVs, except lineage 4 which contained two novel rodent HEVs and known rat HEVs.

#### CoVs

**Fig. 5:**
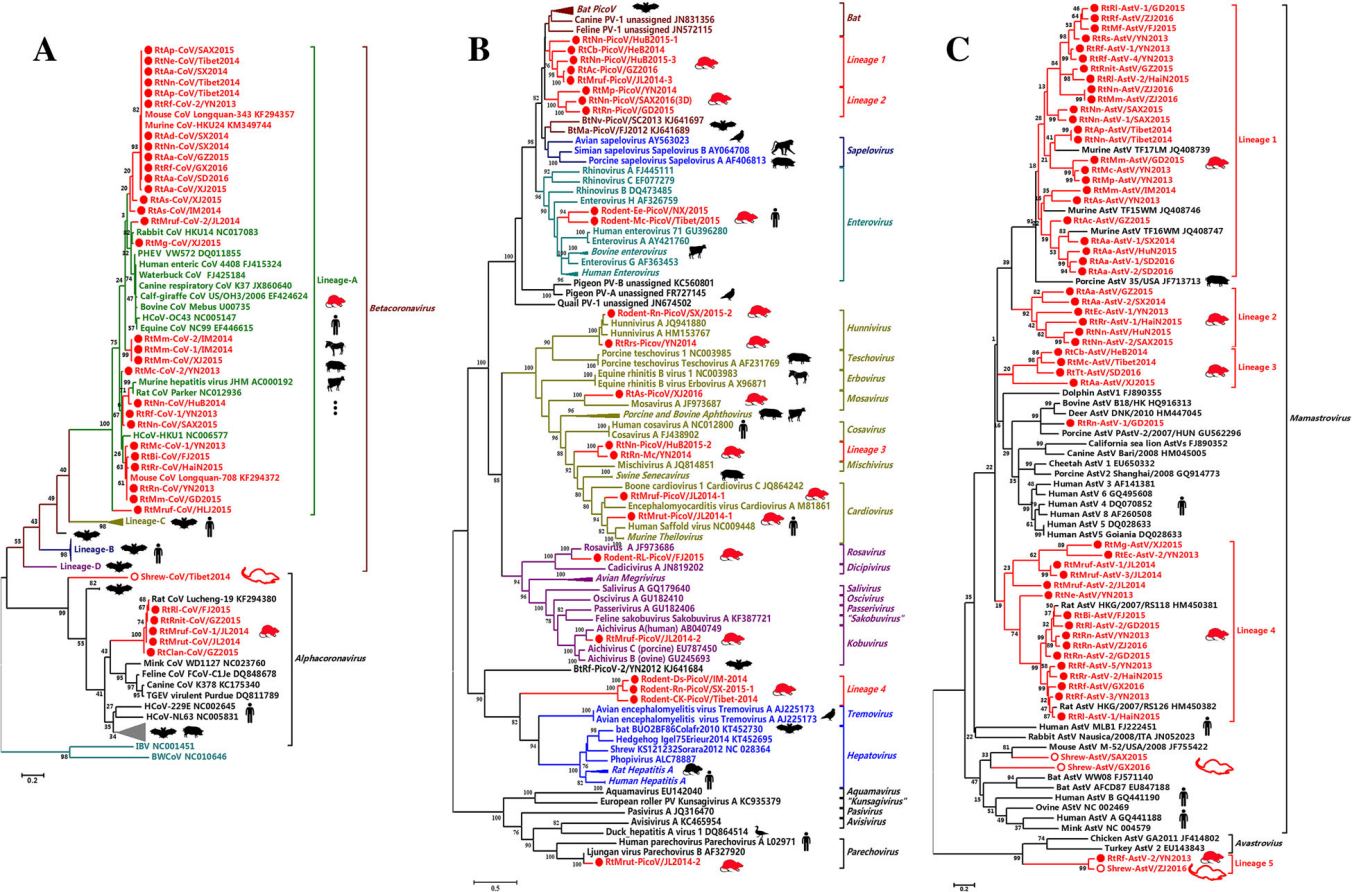
**a** Phylogenetic tree based on the partial RdRp (NSP12) proteins of CoVs. **b** Phylogenetic tree based on the complete RNA-dependent RNA polymerase proteins of PicoVs. **c** Phylogenetic tree based on 387 nucleotides of the partial RdRp gene of AstVs. The viruses found in this study are labeled in red font. The relationships between viruses and their hosts were shown in Additional file 3: Figures S10, S11, and S12

One hundred and eighteen rodent samples and five *Sorex araneus* samples were identified as CoV positive; 35 strains were selected for sequencing of partial RNA-dependent RNA polymerase (RdRp), and 12 strains were characterized for genome sequences (Additional file 1:Table S4). Pairwise similarity and phylogenetic analysis (Fig. 5a and Additional file 3: Figure S6) revealed that 29 viruses formed diverse evolutionary clades in lineage A under the genus *Betacoronavirus*, with sequence identities between 88.1 and 98.9% (RdRp aa identity). The other six viruses were all assigned to the genus *Alphacoronavirus*; five of these clustering as a rodent-borne clade within the *Alphacoronavirus* genus, with sequence identities between 98.4 and 98.8% (RdRp aa identity). One virus, Shrew-CoV/Tibet2014, identified in *Sorex araneus* from Tibet appeared to have undergone separate evolution, phylogenetically distant from all other α-CoVs with < 66.0% RdRp aa identity (Additional file 1: Tables S9 and S10).

#### PicoVs

Sixty rodent samples were identified as PicoV positive, and 23 strains underwent genome sequencing (Additional file 1: Table S4) [14]. Rodent viruses from the genera *Enterovirus*, *Hunnivirus*, *Mosavirus*, *Cardiovirus*, *Rosavirus*, *Kobuvirus*, and *Parechovirus* were found in this study and showed 48.3–56.4%, 80.4–80.8%, 47%, 46.8–60.3%, 60.9%, 63–76.9%, and 43.7–87.3% RdRp aa identities with known members in each genus, respectively (Fig. 5b and Additional file 1: Table S11). Eight viruses formed lineages 1 and 2 close to the bat PicoV clade with 38.1–43.6%, 33.5–38.8%, and 48.2–56.7% aa identities with bat PicoVs in the P1, P2, and P3 regions, respectively. Two novel lineages 3 and 4 were identified with < 10.2–28.9% aa identities in the P1 region, 17.3–23.6% in the P2 region, and 21.8–28.4% in the P3 region compared with other PicoVs (Additional file 1: Table S10). Viruses closely related to known PicoVs of other hosts were found (e.g., rodent viruses related to human aichivirus, human rosavirus, and bovine hunnivirus) [14].

#### AstVs

A larger number of AstVs were detected in both rodent and shrew samples (Additional file 1: Table S4). Fifty-five AstVs were selected for sequencing. Most of the rodent AstVs sequenced belonged to four main genetic lineages 1 to 4 within the genus *Mamastrovirus* and had less sequence similarity with AstVs in other hosts (Fig. 5c). One rodent AstV, RtRn-AstV-1/GD2015, was closely related to AstVs of cattle, deer, and pigs with > 90% nt identity. Two shrew AstVs, Shrew-AstV/SAX2015 and Shrew-AstV/GX2016, were related to mouse AstV with ~ 70% nt identity in the genus *Mamastrovirus*. Lineage 5 contained one shrew AstV and one mouse AstV, with 79% nt identity with each other. Lineage 5 branched out of the genus *Mamastrovirus* and showed a closer relationship with the genus *Avastrovius*.

#### Paramyxoviruses (ParaVs)

All reads of ParaVs from different rodent species were closely related to previously reported Beilong or Tailam viruses [35–37]. We obtained full-length sequence of RtAp-ParaV/NX2015 from *Apodemus peninsulae*, which we assigned to *Jeilongvirus* close to the Beilong and Tailam virus clade (74.2–79.2% aa identities for L) (Additional file 3: Figure S7 and Additional file 1: Table S12).

#### Caliciviruses (CalV)

We characterized Shrew-CalV/Tibet2014 in *Sorex araneus* as a novel species of the genus *Norovirus* with 34.5–51.2% aa identities with known Noroviruses (Additional file 3: Figure S8). The most closely related murine norovirus that was classified as genogroup V showed 51.2% aa identity with this shrew CalV.

### Evolutionary characteristics of DNA viruses

#### Circoviruses (CVs)

**Fig. 6:**
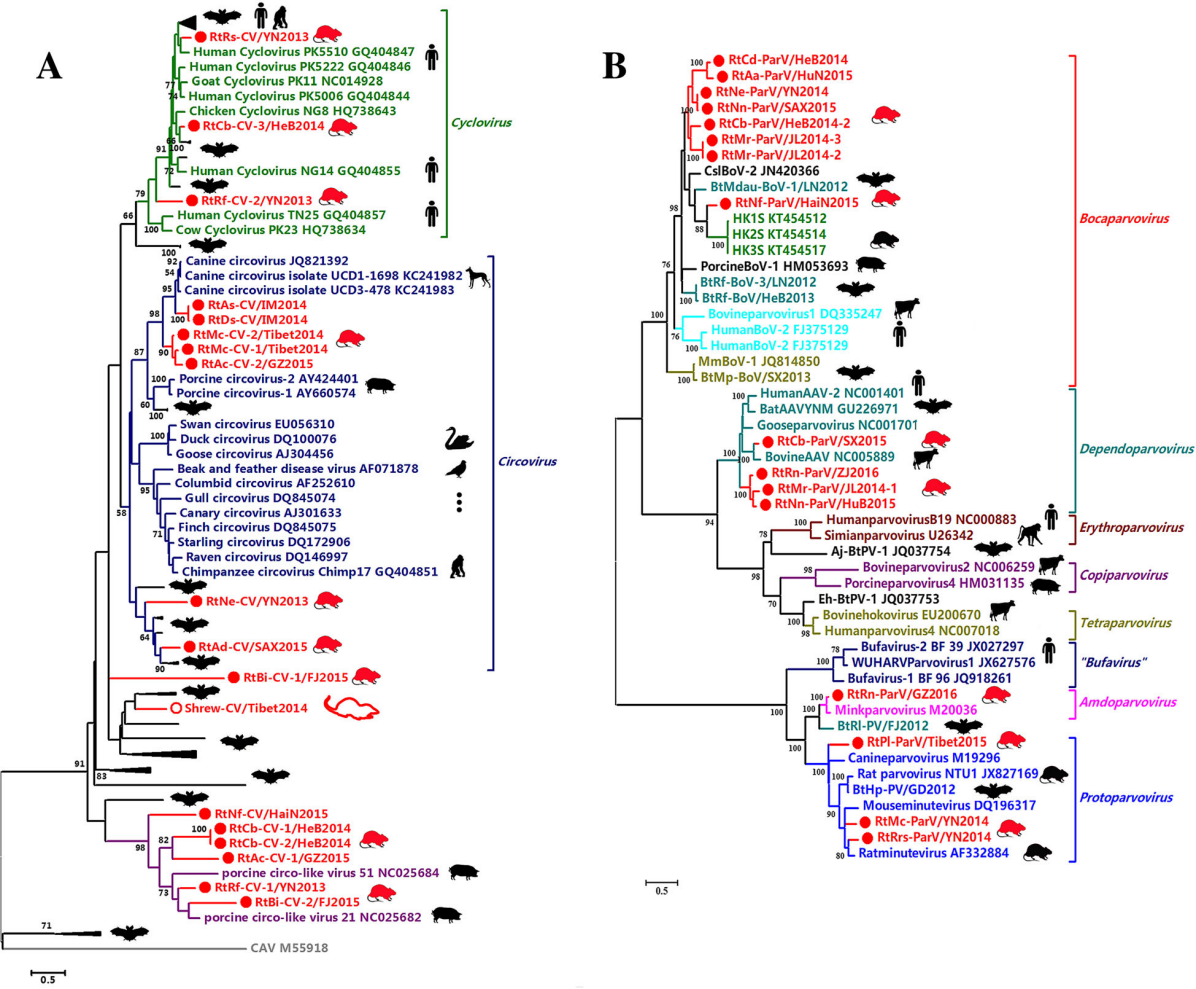
**a** Phylogenetic tree based on the complete replicase (Rep) proteins of CVs. **b** Phylogenetic tree based on the VP1 proteins of members of the subfamily *Parvovirinae*. The viruses found in this study are labeled in red font. The relationships between viruses and their hosts were shown in Additional file 3: Figures S13 and S14

Thirty-three CV-positive samples were confirmed, and 18 CV strains were identified for genome sequencing (Additional file 1: Table S4). Pairwise alignment and phylogenetic analysis suggested that 10 of these belonged to the genera *Circovirus* and *Cyclovirus*. The other eight novel rodent or shrew CVs branched out of the root of the genera *Circovirus* and *Cyclovirus* (Fig. 6a).

#### ParVs

Twenty-eight ParV-positive samples were identified, and 16 virus strains were selected for genome sequencing (Additional file 1: Table S4). These viruses clustered phylogenetically with *Bocaparvovirus*, *Dependoparvovirus*, *Amdoparvovirus*, and *Protoparvovirus* (Fig. 6b). Eight rodent ParVs clustered together and formed a novel lineage of Rodent *Bocavirus* under the genus *Bocaparvovirus*, with < 57.0% aa identity with bocaviruses of other hosts. Three ParVs were classified in the genus *Protoparvovirus* but formed novel evolutionary clades. One ParV was clustered with mink parvovirus (82.7% aa identity) under the genus *Amdoparvovirus*. The other four ParVs formed two lineages under the genus *Dependoparvovirus* with < 63.1% aa identity with known adeno-associated virus (Additional file 1: Table S13).

#### Adenoviruses (AdVs)

Thirty-one samples were AdV positive (Additional file 1: Table S4), and seven strains were selected for sequencing of partial DNA polymerase gene. Two rodent AdVs clustered with previously reported murine AdVs (MAdV-1, MAdV-2, and MAdV-3; 59.7–76.6% aa identities, Additional file 3: Figure S9) [38, 39]. Three rodent AdVs formed a separate genetic lineage within *Mastadenovirus*. Two shrew AdVs were also identified; one closely related to previously reported tree shrew AdV-1 and another located in a novel lineage related to Bat AdV-FBV1.

#### Papillomavirus (PVs)

RtAc-PV/GZ2015 in *Apodemus chevrieri* and RtRn-PV/GD2014 in *Rattus norvegicus* were full-length sequenced (Additional file 1: Table S4). These two rodent viruses showed high sequence similarities with known rodent PVs from *Apodemus sylvaticus* and *Rattus norvegicus* in Germany (90% and 100% aa identities) [40, 41].

### Tanglegram comparing the evolutions between viruses and hosts

When we mapped the viral phylogram to the evolutionary lineages of their hosts (Figs. 3 and 4, Additional file 3: Figures S10-S14), co-evolution or co-divergence between viruses and their rodent and insectivore hosts were observed in most cases such as HVs, AreVs, ArteVs, and HEVs. Most viruses in each family tended to form different lineages that were phylogenetically consistent with the phylogeny of their hosts from species to family. For example, the division of HV phylogroups was congruent with the phylogenies of their *Arvicolinae*, *Sigmodontinae*, *Murinae*, and *Soricidae* hosts; the separation of AreVs in the old world complex was congruent with the phylogenies of their *Murinae* and *Dipodidae* hosts. However, virus phylogeny was not always consistent with host phylogeny, and multiple incongruous relationships between the phylogenies of hosts and viruses were also found. For example, *Dipus sagitta* and RtDs-HV/IM2014, *Rattus tanezumi* and RtRf-AstV-2/YN2013, *Cricetulus* species and their HEVs, and rodent species and many of their hepaciviruses and PicoVs were phylogenetically incongruent. These suggested that host shifts seem to be common for several virus phylogenetic lineages.

## Discussion

Novel EIDs from animal reservoirs pose an increasing threat to global health and security. Comprehensive knowledge of the viral population and ecological community resident in animal reservoirs, especially in wildlife, could minimize the impact of potential animal-originated EIDs on public health by providing meaningful basic data [3, 32]. A predictive analysis based on host and viral traits conducted by Olival et al. revealed that the present observed viral richness of wildlife is limited, and there is still an alarming number of “missing viruses” and “missing zoonotic viruses” that merit further systematic surveillance globally, especially in rodents, primates, and bats [7].

By firstly characterizing the pharyngeal and anal viromes of representative rodents and other small mammals throughout China, the present study identified many novel viruses from rodents and insectivores. When added to our recent work on bats [31, 32], the present results suggest that there is rich as-yet-undiscovered viral diversity in rodents, bats, insectivores, and other mammalian species in China. Viral diversity among different mammalian hosts is strikingly different. The presence of a large number of diverse RNA and DNA viruses with high prevalence and abundance highlights that both rodents and soricomorphs can tolerate diverse viruses, as we described previously in bats [32]. Animals in northern and western regions show similar or even higher viral diversity when compared with those of animals in central and southern areas of China. A recent report showed that Central and Southern China with higher human population density may have a high estimated risk of zoonotic EID events based on previous data [42]. Here, the identification of diverse viruses and even pathogen-related viruses in Northern and Western China indicates that the risk of EIDs originating from wildlife in these regions should not be underestimated.

In addition to the viral family-specific findings discussed below, our study has some potentially broad implications. First, we have characterized diverse HVs, AreVs, aeteriviruses, picobirnaviruses, CoVs, hepaciviruses, PestVs, PicoVs, HEVs, AstVs, herpesviruses, anelloviruses, AdVs, CVs, and ParVs, extending our knowledge for members of each viral family, and identifying new viral genera and species, albeit that the role of some viruses in human illness is unclear [43, 44]. Second, we showed that rodent species carry more viruses than previously thought (e.g., identification of viruses of the genera *Bocaparvovirus* and *Pestivirus*), extending the known host range of the viral family and suggesting that rodents should be considered potential carriers and disseminators of viruses in these genera or species. Third, we also found that many viruses (e.g., AreVs, CoVs, and PicoVs) identified from the same or different animal species from different locations shared high sequence identities and close genetic relationships; the phylogenetic relationships we uncovered suggest these rodent- and shrew-borne viruses have the potential for intra- or cross-species transmission concomitant with intra- or inter-species contact and then co-evolve with their hosts in a wide geographical area. Finally, the phylogenetic incongruence between hosts and their viruses in many cases suggests that host shifts have happened frequently during the viral evolutionary history, which may create opportunities for the emergence of new viruses that are able to adapt to new hosts.

Rodent origin HVs and AreVs are important causative agents of human hemorrhagic fever and related diseases [8]. Although prevention and control measures have been performed in recent years, hemorrhagic fever with renal syndrome (HFRS) caused by HVs remains a serious public health problem in China, and the number of HFRS cases still accounts for ~ 90% of the total cases worldwide [13, 24, 45]. Although many members of the genus *Mammarenavirus* in the family *Arenaviridae* are associated with human diseases worldwide, only LCMV and WENV have been reported in China [11, 46]. Although some cases of lymphocytic choriomeningitis were clinically diagnosed in China based on serology and pathology, LCMV has not been laboratory confirmed nor full-length sequenced in China. We identified diverse HVs and AreVs located in different phylogenetic positions that indicate the presence of novel viruses in new hosts such as *Caryomys eva* (RtCe-HV/NX2015), *Eothenomys melanogaster* (RtCl-HV/GZ2015), and *Dipus sagitta* (RtDs-HV/IM2014 and RtDs-AreV/IM2014). These findings are important for the prevention of HVs and AreVs transmitted from rodents. We also identified novel AreVs in different *Rattus* species with high sequence identities from Yunnan, Hunan, and Zhejiang provinces. These *Rattus* species hosts have high potential for contact with people in rural China, and the clustering of these viruses from different provinces with WENV variants identified in humans suggests there is a broader geographical distribution of these potentially zoonotic viruses [11, 47].

We identified diverse rodent ArteVs phylogenetically scattered throughout the *Arteriviridae*, and with higher genetic diversity than ArteVs of other host groups. This suggests that rodents are important wildlife hosts for a variety of ArteVs species with different evolutionary stages and that rodents may act as the main wildlife reservoirs of ArteVs. Infection by the ArteV PRRSV causes severe disease in swine and global economic losses to the swine industry [48, 49]. All previously reported PRRSV strains are only detected in swine and can be phylogenetically classified into genotype 1 (or European type, prototype: Lelystad virus) and genotype 2 (or North American type, prototype: VR-2332) without any intermediate genotype [50, 51]. Our characterization of four rodent ArteVs in the PRRSV species phylogenetically clustered between the two known genotypes suggests that the virus evolved independently in pigs and rats and finally formed at least three different genotypes under the PRRSV species. It also indicates the presence of a closer ancestor of PRRSV in rodents than the previously hypothesized mouse LDV.

Unlike bat CoVs which are grouped into various evolutionary clades of the genera *Alphacoronavirus* and *Betacoronavirus* [32, 52–55], CoVs identified in various rodent species from various regions can only be classified into two lineages: lineage A of *Betacoronavirus* and a separate lineage of *Alphacoronavirus*. Human CoV OC43, human CoV HKU1, and bovine CoV under lineage A of *Betacoronavirus* are human or animal pathogens that cause mild-to-severe diseases [28]. The identification of diverse lineage-A rodent β-CoVs confirms the hypothesis that rodents may be an important reservoir for ancestors of lineage-A β-CoVs [28, 29]. The identification of α-CoVs under separate lineage from diverse provinces suggests a broader geographical distribution of these rodent-specific viruses.

Although samples from individuals of the order *Lagomorpha* and *Soricomorpha* were limited, many novel viruses were identified from *Soricomorpha* insectivores, a group of mammals that is poorly sampled, but abundant in natural and human-dominated habitats. The characterization of a shrew-borne CoV (Shrew-CoV/Tibet2014), CalV (Shrew-CalV/Tibet2014), AstVs, and CVs (Shrew-CV/Tibet2014) and a distinctive lineage of the genera *Alphacoronavirus* and *Norovirus* indicates that these insectivores may harbor a diversity of these and other viruses; some of which may have zoonotic potential.

## Conclusions

These findings, combined with our previous bat virome data, greatly increase our knowledge of the viral community in wildlife in a densely populated country in an emerging disease hotspot. Continued efforts in viral discovery in these and other mammalian hosts in China may reveal greater diversity of viral lineages, as shown recently for arthropods [56, 57], and as hypothesized globally [58].

## Methods

### Animal samples

Collection of animal samples was conducted within the National HFRS Surveillance Network and National Medical Vectors Surveillance Network. Capture was conducted using live traps supplied by the State Key Laboratory for Infectious Diseases Prevention and Control. Most animals captured were euthanized by carbon dioxide although some individuals of rare species (labeled in red with IUCN red list level in Additional file 1: Table S1) were released after sampling. Pharyngeal and anal swabs were placed in virus sampling tubes (Yocon, Beijing, China) containing maintenance medium and temporarily stored at − 20 °C. After the sampling was finished, samples were transported to the laboratory and stored at − 80 °C. Samples from the same species and from the same site were pooled. The voucher collection of specimens was deposited at − 80 °C by the Department of Vector Biology and Control, National Institute for Communicable Disease Control and Prevention, Chinese Center for Disease Control and Prevention.

### Viral nucleic acid library construction, next-generation sequencing, and taxonomic assignments

Swab samples in maintenance medium were re-suspended, processed with a viral particle-protected nucleic acid purification method, and amplified by sequence-independent RT-PCR as described previously [32]. Briefly, the samples were filtered through a 0.45-μm polyvinylidene difluoride filter (Millipore, Darmstadt, Germany) to remove eukaryotic and bacterium-sized particles. The filtered samples were then centrifuged at 150,000×*g* for 3 h at 4 °C. The pellets were re-suspended in Hank’s balanced salt solution. To remove naked DNA and RNA, the re-suspended pellet was digested in a cocktail of DNase and RNase enzymes, including Turbo DNase (Ambion, Austin, TX, USA), benzonase (Novagen, Darmstadt, Germany), and RNase One (Promega, Madison, WI, USA) at 37 °C for 2 h. The viral DNA and RNA were simultaneously isolated using a QIAmp MinElute Virus Spin Kit (Qiagen, Valencia, CA, USA). Viral first-strand cDNA was synthesized using the primer K-8N and a Superscript III system (Invitrogen, Carlsbad, CA, USA)*.* To convert first-strand cDNA into dsDNA, the cDNA was incubated at 37 °C for 1 h in the presence of Klenow fragment (NEB, Ipswich, MA, USA). Sequence-independent PCR amplification was conducted using primer K and Phusion DNA polymerase (NEB). The PCR products were analyzed by agarose gel electrophoresis. All DNA smears larger than 500 bp were excised and extracted with a MinElute Gel Extraction Kit (Qiagen).

Amplified viral nucleic acid libraries were analyzed using an Illumina HiSeq2500 sequencer (Illumina, San Diego, CA, USA) for a single read of 100 bp in length. Raw sequence reads were filtered using previously described criteria to obtain valid sequences [31, 32, 59], reads with no call sites, reads with similarity to the sequencing adaptor, and the primer K sequence, and duplicate reads and low-complexity reads were culled. Each read was evaluated for viral origin by conducting alignments with the NCBI non-redundant nucleotide database (NT) and protein database (NR) using BLASTn and BLASTx (with parameters -e 1e-5 –F T). Reads with no hits in NT or NR were further assembled by metagenomics assemblers (e.g., MetaVelvet, IDBA-UD, and SOAPdenovo), and the contigs were again aligned with NT and NR to identify any viruses present. Taxonomy of the aligned reads with the best BLAST scores (*E* value < 10^−5^) from all lanes was parsed and exported with MEGAN 4—MetaGenome Analyzer [60]. We also tested an assembly-first strategy to analyze sequence data. Reads were first assembled by metagenomics assemblers, and output contigs were then aligned to NT and NR.

### Calculating viral prevalence

Sequence reads classified as the same virus family or genus by MEGAN 4 were extracted and assembled with SeqMan program (Lasergene; DNAstar, Madison, WI, USA). The accurate locations of the assembled reads and the relative distances between them were determined. A draft genome with several SNPs of each virus was obtained. Based on partial genomic sequences of viruses obtained by assembly, we designed specific nested primers for PCR and RT-PCR to screen for each virus in individual samples from each mammalian species. Different degenerate primers targeting conserved regions were also used to identify the presence and prevalence of viruses [12, 61–66].

### Genome sequencing

Locations of reads and the distances between reads of the same virus were determined using alignment results exported by MEGAN 4. Representative positive samples for each virus were selected for genome sequencing. Reads with accurate genomic locations were then used for reads-based PCR to identify partial genomes. Based on the partial genomic sequences obtained by specific nested PCR, the remaining genomic sequences were determined using inverse PCR, genome walking, and 5′- and 3′- rapid amplification of cDNA ends (RACE).

### Genomic and phylogenetic analysis

Nucleotide sequences of genomes and amino acid sequences of open reading frames (ORFs) were deduced by comparing them with other viral sequences. The conserved protein families and domains were predicted using Pfam and InterProScan 5 (available at: http://www.ebi.ac.uk/services/proteins). Routine sequence alignments were performed using Clustal Omega, Needle (available at: http://www.ebi.ac.uk/Tools/), MegAlign (Lasergene), and T-coffee with manual curation. MEGA6.0 (Phoenix, AZ, USA) was used to align the nt and the deduced aa sequences using the MUSCLE package and default parameters. The best substitution model was evaluated using the Model Selection package. We used maximum-likelihood to process the phylogenetic analyses with 1,000 bootstrap replicates [67]. The aa identities and genetic distances were calculated using the ML method with a pairwise evolutionary distance calculation as the distance metric. The evolutionary lineages of involved hosts were drawn based on mitochondrial cytochrome *b* (mt-*cyt b*) according to previous reports [7, 9, 68–70]. The congruence between the phylogenies of viruses and their hosts were determined by tanglegram which is generated by matching each virus to their associated host [12, 71].

### Nucleotide sequence accession numbers

All genome sequences were submitted to GenBank (accession numbers are given in Additional file 1: Table S4). The Illumina HiSeq2500 sequence data were deposited into the NCBI sequence reads archive (SRA) under accession number PRJNA375958.

#### Description of supplementary information (SI)

Supplementary tables and figures are available with the online version of this paper.

## Additional files

Additional file 1: **Table S1.** Samples of the 55 animal species used in this study and the provinces and dates of collection. **Table S2.** The reads of virus under each family. **Table S3.** The reads of mammailan virus under each family. **Table S4.** Origin and accession number of viruses identified in this study. **Table S5.** Amino acid identity of rodents Arenavirus and representatives of other species. **Table S6.** The aa identities (%) of the predicted ORF1a and ORF1b between Rodent Arteriviruses and other known members of the family Arteriviridae (including a tentative member, WPDV). **Table S7.** The aa identities (%) between these hepaciviruses and other known rodent hepacivirus. **Table S8.** Amino acid identity (%)of rodents Hepatitis E and representatives of other Genotypes. **Table S9.** The aa identities (%) between these BtCoVs and other known members of the lineage-A beta-CoVs. **Table S10.** The aa identities (%) between these BtCoVs and other known members of alpha-CoVs. **Table S11.** Amino acid identity of rodents picornaviruses and representatives of other genera in P1, P2, and P3 regions. **Table S12.** Pairwise amino acid identities (%) of predicted gene products of RtAp-ParaV/NX2015 compared to other Jeilongvirus members. **Table S13.** Amino acid identity of rodents parvovirus and representatives of other genera. (XLSX 149 kb)

Additional file 2: **Table S14.** The reads of each viral family related to province and animal species. (XLSX 159 kb)

Additional file 3: **Figure S1.** Arenavirus G. Phylogenetic tree showing the relationships (amino acid) between arenaviruses in the G polymerase. The viruses found in this study are labeled in red font. **Figure S2.** Arenavirus N. Phylogenetic tree showing the relationships (amino acid) between arenaviruses in the N polymerase. The viruses found in this study are labeled in red font. **Figure S3.** Phylogenetic tree showing the relationships (amino acid) between arteriviruses in the pp1a proteins. The viruses found in this study are labeled in red font. **Figure S4.** Genomic organization of the Rat-arterivirus-1/Ningxia2015 and Rat-arterivirus/Jilin2014. Figure S5. phylogenetic tree based on the polyproteins of TBEV. The viruses found in this study are labeled in red font. **Figure S6.** Phylogenetic tree based on the complete Spike (S) proteins of CoVs. The viruses found in this study are labeled in red font. **Figure S7.** Phylogenetic treebased on the L proteins of ParaVs. The viruses found in this study are labeled in red font. **Figure S8.** Phylogenetic tree based on the polyproteins of Noroviruses. The viruses found in this study are labeled in red font. **Figure S9.** Phylogenetic tree based on diverse sequences of partial amino acid of the polymerases of AdVs. The viruses found in this study are labeled in red font. **Figure S10.** The phylogenetic relationships between CoVs and their hosts. **Figure S11.** The phylogenetic relationships between PicoVs and their hosts. **Figure S12.** The phylogenetic relationships between AstVs and their hosts. **Figure S13.** The phylogenetic relationships between CVs and their hosts. **Figure S14.** The phylogenetic relationships between ParVs and their hosts. (DOCX 5298 kb)

## References

[1] Jones KE, Patel NG, Levy MA, Storeygard A, Balk D, Gittleman JL, Daszak P (2008). Global trends in emerging infectious diseases. Nature.

[2] Taylor LH, Latham SM, Woolhouse ME (2001). Risk factors for human disease emergence. Philos Trans R Soc Lond Ser B Biol Sci.

[3] Wu Tong, Perrings Charles, Kinzig Ann, Collins James P., Minteer Ben A., Daszak Peter (2016). Economic growth, urbanization, globalization, and the risks of emerging infectious diseases in China: A review. Ambio.

[4] Wolfe ND, Dunavan CP, Diamond J (2007). Origins of major human infectious diseases. Nature.

[5] Lloyd-Smith JO, George D, Pepin KM, Pitzer VE, Pulliam JR, Dobson AP, Hudson PJ, Grenfell BT (2009). Epidemic dynamics at the human-animal interface. Science.

[6] Smith I, Wang LF (2013). Bats and their virome: an important source of emerging viruses capable of infecting humans. Curr Opin Virol.

[7] Olival KJ, Hosseini PR, Zambrana-Torrelio C, Ross N, Bogich TL, Daszak P (2017). Host and viral traits predict zoonotic spillover from mammals. Nature.

[8] Meerburg BG, Singleton GR, Kijlstra A (2009). Rodent-borne diseases and their risks for public health. Crit Rev Microbiol.

[9] Blanga-Kanfi S, Miranda H, Penn O, Pupko T, DeBry RW, Huchon D (2009). Rodent phylogeny revised: analysis of six nuclear genes from all major rodent clades. BMC Evol Biol.

[10] Wilson DE, Reeder DM (2005). Mammal species of the world : a taxonomic and geographic reference.

[11] Li K, Lin XD, Wang W, Shi M, Guo WP, Zhang XH, Xing JG, He JR, Wang K, Li MH (2015). Isolation and characterization of a novel arenavirus harbored by rodents and shrews in Zhejiang province, China. Virology.

[12] Guo WP, Lin XD, Wang W, Tian JH, Cong ML, Zhang HL, Wang MR, Zhou RH, Wang JB, Li MH (2013). Phylogeny and origins of hantaviruses harbored by bats, insectivores, and rodents. PLoS Pathog.

[13] Cao S, Ma J, Cheng C, Ju W, Wang Y (2016). Genetic characterization of hantaviruses isolated from rodents in the port cities of Heilongjiang, China, in 2014. BMC Vet Res.

[14] Du J, Lu L, Liu F, Su H, Dong J, Sun L, Zhu Y, Ren X, Yang F, Guo F (2016). Distribution and characteristics of rodent picornaviruses in China. Sci Rep.

[15] Firth C, Bhat M, Firth MA, Williams SH, Frye MJ, Simmonds P, Conte JM, Ng J, Garcia J, Bhuva NP (2014). Detection of zoonotic pathogens and characterization of novel viruses carried by commensal Rattus norvegicus in New York City. MBio.

[16] Phan TG, Kapusinszky B, Wang C, Rose RK, Lipton HL, Delwart EL (2011). The fecal viral flora of wild rodents. PLoS Pathog.

[17] Fehér Enikő, Kemenesi Gábor, Oldal Miklós, Kurucz Kornélia, Kugler Renáta, Farkas Szilvia L., Marton Szilvia, Horváth Győző, Bányai Krisztián, Jakab Ferenc (2016). Isolation and complete genome characterization of novel reassortant orthoreovirus from common vole (Microtus arvalis). Virus Genes.

[18] Han BA, Schmidt JP, Bowden SE, Drake JM (2015). Rodent reservoirs of future zoonotic diseases. Proc Natl Acad Sci U S A.

[19] Palacios G, Druce J, Du L, Tran T, Birch C, Briese T, Conlan S, Quan PL, Hui J, Marshall J (2008). A new arenavirus in a cluster of fatal transplant-associated diseases. N Engl J Med.

[20] Briese T, Paweska JT, McMullan LK, Hutchison SK, Street C, Palacios G, Khristova ML, Weyer J, Swanepoel R, Egholm M (2009). Genetic detection and characterization of Lujo virus, a new hemorrhagic fever-associated arenavirus from southern Africa. PLoS Pathog.

[21] Charrel RN, de Lamballerie X (2010). Zoonotic aspects of arenavirus infections. Vet Microbiol.

[22] Ishii A, Thomas Y, Moonga L, Nakamura I, Ohnuma A, Hang'ombe B, Takada A, Mweene A, Sawa H (2011). Novel arenavirus, Zambia. Emerg Infect Dis.

[23] Zhang YZ, Zhang FX, Wang JB, Zhao ZW, Li MH, Chen HX, Zou Y, Plyusnin A (2009). Hantaviruses in rodents and humans, Inner Mongolia Autonomous Region, China. Emerg Infect Dis.

[24] Zhang S, Wang S, Yin W, Liang M, Li J, Zhang Q, Feng Z, Li D (2014). Epidemic characteristics of hemorrhagic fever with renal syndrome in China, 2006-2012. BMC Infect Dis.

[25] Valarcher JF, Hagglund S, Juremalm M, Blomqvist G, Renstrom L, Zohari S, Leijon M, Chirico J (2015). Tick-borne encephalitis. Rev Sci Tech.

[26] Drexler JF, Corman VM, Lukashev AN, van den Brand JM, Gmyl AP, Brunink S, Rasche A, Seggewibeta N, Feng H, Leijten LM (2015). Evolutionary origins of hepatitis A virus in small mammals. Proc Natl Acad Sci U S A.

[27] Drexler JF, Corman VM, Muller MA, Lukashev AN, Gmyl A, Coutard B, Adam A, Ritz D, Leijten LM, van Riel D (2013). Evidence for novel hepaciviruses in rodents. PLoS Pathog.

[28] Lau SK, Woo PC, Li KS, Tsang AK, Fan RY, Luk HK, Cai JP, Chan KH, Zheng BJ, Wang M, Yuen KY (2015). Discovery of a novel coronavirus, China Rattus coronavirus HKU24, from Norway rats supports the murine origin of Betacoronavirus 1 and has implications for the ancestor of Betacoronavirus lineage A. J Virol.

[29] Wang W, Lin XD, Guo WP, Zhou RH, Wang MR, Wang CQ, Ge S, Mei SH, Li MH, Shi M (2015). Discovery, diversity and evolution of novel coronaviruses sampled from rodents in China. Virology.

[30] Wang Y (2003). A complete checklist of mammal species and subspecies in China: a taxonomic and geographic reference.

[31] Wu Z, Ren X, Yang L, Hu Y, Yang J, He G, Zhang J, Dong J, Sun L, Du J (2012). Virome analysis for identification of novel mammalian viruses in bat species from Chinese provinces. J Virol.

[32] Wu Z, Yang L, Ren X, He G, Zhang J, Yang J, Qian Z, Dong J, Sun L, Zhu Y (2016). Deciphering the bat virome catalog to better understand the ecological diversity of bat viruses and the bat origin of emerging infectious diseases. ISME J.

[33] Chen L, Liu B, Yang J, Jin Q (2014). DBatVir: the database of bat-associated viruses. Database (Oxford).

[34] Chen L, Liu B, Wu Z, Jin Q, Yang J (2017). DRodVir: a resource for exploring the virome diversity in rodents. J Genet Genomics.

[35] Woo PC, Lau SK, Wong BH, Wong AY, Poon RW, Yuen KY (2011). Complete genome sequence of a novel paramyxovirus, Tailam virus, discovered in Sikkim rats. J Virol.

[36] Li Z, Yu M, Zhang H, Magoffin DE, Jack PJ, Hyatt A, Wang HY, Wang LF (2006). Beilong virus, a novel paramyxovirus with the largest genome of non-segmented negative-stranded RNA viruses. Virology.

[37] Jack PJ, Boyle DB, Eaton BT, Wang LF (2005). The complete genome sequence of J virus reveals a unique genome structure in the family Paramyxoviridae. J Virol.

[38] Klempa B, Kruger DH, Auste B, Stanko M, Krawczyk A, Nickel KF, Uberla K, Stang A (2009). A novel cardiotropic murine adenovirus representing a distinct species of mastadenoviruses. J Virol.

[39] Hemmi S, Vidovszky MZ, Ruminska J, Ramelli S, Decurtins W, Greber UF, Harrach B (2011). Genomic and phylogenetic analyses of murine adenovirus 2. Virus Res.

[40] Schulz E, Gottschling M, Ulrich RG, Richter D, Stockfleth E, Nindl I (2012). Isolation of three novel rat and mouse papillomaviruses and their genomic characterization. PLoS One.

[41] Schulz E, Gottschling M, Wibbelt G, Stockfleth E, Nindl I (2009). Isolation and genomic characterization of the first Norway rat (Rattus norvegicus) papillomavirus and its phylogenetic position within Pipapillomavirus, primarily infecting rodents. J Gen Virol.

[42] Allen T, Murray KA, Zambrana-Torrelio C, Morse SS, Rondinini C, Di Marco M, Breit N, Olival KJ, Daszak P (2017). Global hotspots and correlates of emerging zoonotic diseases. Nat Commun.

[43] Ganesh B, Masachessi G, Mladenova Z (2014). Animal picobirnavirus. Virusdisease.

[44] Manzin A, Mallus F, Macera L, Maggi F, Blois S (2015). Global impact of Torque teno virus infection in wild and domesticated animals. J Infect Dev Ctries.

[45] Manigold T, Vial P (2014). Human hantavirus infections: epidemiology, clinical features, pathogenesis and immunology. Swiss Med Wkly.

[46] Morita C, Tsuchiya K, Ueno H, Muramatsu Y, Kojimahara A, Suzuki H, Miyashita N, Moriwaki K, Jin ML, Wu XL, Wang FS (1996). Seroepidemiological survey of lymphocytic choriomeningitis virus in wild house mice in China with particular reference to their subspecies. Microbiol Immunol.

[47] Blasdell KR, Duong V, Eloit M, Chretien F, Ly S, Hul V, Deubel V, Morand S, Buchy P (2016). Evidence of human infection by a new mammarenavirus endemic to Southeastern Asia. Elife.

[48] Nilubol D, Tripipat T, Hoonsuwan T, Kortheerakul K (2012). Porcine reproductive and respiratory syndrome virus, Thailand, 2010-2011. Emerg Infect Dis.

[49] An TQ, Tian ZJ, Leng CL, Peng JM, Tong GZ (2011). Highly pathogenic porcine reproductive and respiratory syndrome virus, Asia. Emerg Infect Dis.

[50] Nelsen CJ, Murtaugh MP, Faaberg KS (1999). Porcine reproductive and respiratory syndrome virus comparison: divergent evolution on two continents. J Virol.

[51] Brar MS, Shi M, Hui RK, Leung FC (2014). Genomic evolution of porcine reproductive and respiratory syndrome virus (PRRSV) isolates revealed by deep sequencing. PLoS One.

[52] Yang L, Wu Z, Ren X, Yang F, He G, Zhang J, Dong J, Sun L, Zhu Y, Du J (2013). Novel SARS-like betacoronaviruses in bats, China, 2011. Emerg Infect Dis.

[53] Yang L, Wu Z, Ren X, Yang F, Zhang J, He G, Dong J, Sun L, Zhu Y, Zhang S, Jin Q (2014). MERS-related betacoronavirus in Vespertilio superans bats, China. Emerg Infect Dis.

[54] Du J, Yang L, Ren X, Zhang J, Dong J, Sun L, Zhu Y, Yang F, Zhang S, Wu Z, Jin Q (2016). Genetic diversity of coronaviruses in Miniopterus fuliginosus bats. Sci China Life Sci.

[55] Wu Z, Yang L, Ren X, Zhang J, Yang F, Zhang S, Jin Q (2016). ORF8-related genetic evidence for Chinese horseshoe bats as the source of human severe acute respiratory syndrome coronavirus. J Infect Dis.

[56] Li CX, Shi M, Tian JH, Lin XD, Kang YJ, Chen LJ, Qin XC, Xu J, Holmes EC, Zhang YZ. Unprecedented genomic diversity of RNA viruses in arthropods reveals the ancestry of negative-sense RNA viruses. Elife. 2015;4. 10.7554/eLife.05378. 10.7554/eLife.05378 PMC4384744 25633976

[57] Shi Mang, Lin Xian-Dan, Tian Jun-Hua, Chen Liang-Jun, Chen Xiao, Li Ci-Xiu, Qin Xin-Cheng, Li Jun, Cao Jian-Ping, Eden John-Sebastian, Buchmann Jan, Wang Wen, Xu Jianguo, Holmes Edward C., Zhang Yong-Zhen (2016). Redefining the invertebrate RNA virosphere. Nature.

[58] Carroll D, Daszak P, Wolfe ND, Gao GF, Morel CM, Morzaria S, Pablos-Mendez A, Tomori O, Mazet JAK (2018). The global virome project. Science.

[59] Yang J, Yang F, Ren L, Xiong Z, Wu Z, Dong J, Sun L, Zhang T, Hu Y, Du J (2011). Unbiased parallel detection of viral pathogens in clinical samples by use of a metagenomic approach. J Clin Microbiol.

[60] Huson DH, Mitra S, Ruscheweyh HJ, Weber N, Schuster SC (2011). Integrative analysis of environmental sequences using MEGAN4. Genome Res.

[61] Woo PC, Lau SK, Li KS, Poon RW, Wong BH, Tsoi HW, Yip BC, Huang Y, Chan KH, Yuen KY (2006). Molecular diversity of coronaviruses in bats. Virology.

[62] Tang XC, Zhang JX, Zhang SY, Wang P, Fan XH, Li LF, Li G, Dong BQ, Liu W, Cheung CL (2006). Prevalence and genetic diversity of coronaviruses in bats from China. J Virol.

[63] Chu DK, Poon LL, Guan Y, Peiris JS (2008). Novel astroviruses in insectivorous bats. J Virol.

[64] Li Y, Ge X, Zhang H, Zhou P, Zhu Y, Zhang Y, Yuan J, Wang LF, Shi Z (2010). Host range, prevalence, and genetic diversity of adenoviruses in bats. J Virol.

[65] Wu Z, Yang L, Yang F, Ren X, Jiang J, Dong J, Sun L, Zhu Y, Zhou H, Jin Q (2014). Novel Henipa-like virus, Mojiang paramyxovirus, in rats, China, 2012. Emerg Infect Dis.

[66] Zheng XY, Qiu M, Ke XM, Guan WJ, Li JM, Huo ST, Chen SW, Zhong XS, Zhou W, Xiong YQ (2016). Detection of novel adenoviruses in fecal specimens from rodents and shrews in southern China. Virus Genes.

[67] Tamura K, Stecher G, Peterson D, Filipski A, Kumar S (2013). MEGA6: molecular evolutionary genetics analysis version 6.0. Mol Biol Evol.

[68] Bininda-Emonds OR, Cardillo M, Jones KE, MacPhee RD, Beck RM, Grenyer R, Price SA, Vos RA, Gittleman JL, Purvis A (2007). The delayed rise of present-day mammals. Nature.

[69] Jansa SA, Weksler M (2004). Phylogeny of muroid rodents: relationships within and among major lineages as determined by IRBP gene sequences. Mol Phylogenet Evol.

[70] Meredith RW, Janecka JE, Gatesy J, Ryder OA, Fisher CA, Teeling EC, Goodbla A, Eizirik E, Simao TL, Stadler T (2011). Impacts of the Cretaceous Terrestrial Revolution and KPg extinction on mammal diversification. Science.

[71] Cui J, Han N, Streicker D, Li G, Tang X, Shi Z, Hu Z, Zhao G, Fontanet A, Guan Y (2007). Evolutionary relationships between bat coronaviruses and their hosts. Emerg Infect Dis.

