# Supplementary material for: Comparative analysis of rodent and small mammal viromes to better understand the wildlife origin of emerging infectious diseases
Source: Microbiome. 2018 Oct 3;6:178. doi: 10.1186/s40168-018-0554-9 (PMC6171170; doi:10.1186/s40168-018-0554-9)
Supplement: Supplementary file 3 — Figure S1. Arenavirus G. Phylogenetic tree showing the relationships (amino acid) between arenaviruses in the G polymerase. The viruses found in this study are labeled in red font. Figure S2. Arenavirus N. Phylogenetic tree showing the relationships (amino acid) between arenaviruses in the N polymerase. The viruses found in this study are labeled in red font. Figure S3. Phylogenetic tree showing the relationships (amino acid) between arteriviruses in the pp1a proteins. The viruses found in this study are labeled in red font. Figure S4. Genomic organization of the Rat-arterivirus-1/Ningxia2015 and Rat-arterivirus/Jilin2014. Figure S5. phylogenetic tree based on the polyproteins of TBEV. The viruses found in this study are labeled in red font. Figure S6. Phylogenetic tree based on the complete Spike (S) proteins of CoVs. The viruses found in this study are labeled in red font. Figure S7. Phylogenetic treebased on the L proteins of ParaVs. The viruses found in this study are labeled in red font. Figure S8. Phylogenetic tree based on the polyproteins of Noroviruses. The viruses found in this study are labeled in red font. Figure S9. Phylogenetic tree based on diverse sequences of partial amino acid of the polymerases of AdVs. The viruses found in this study are labeled in red font. Figure S10. The phylogenetic relationships between CoVs and their hosts. Figure S11. The phylogenetic relationships between PicoVs and their hosts. Figure S12. The phylogenetic relationships between AstVs and their hosts. Figure S13. The phylogenetic relationships between CVs and their hosts. Figure S14. The phylogenetic relationships between ParVs and their hosts. (DOCX 5298 kb) [file 40168_2018_554_MOESM3_ESM.docx]

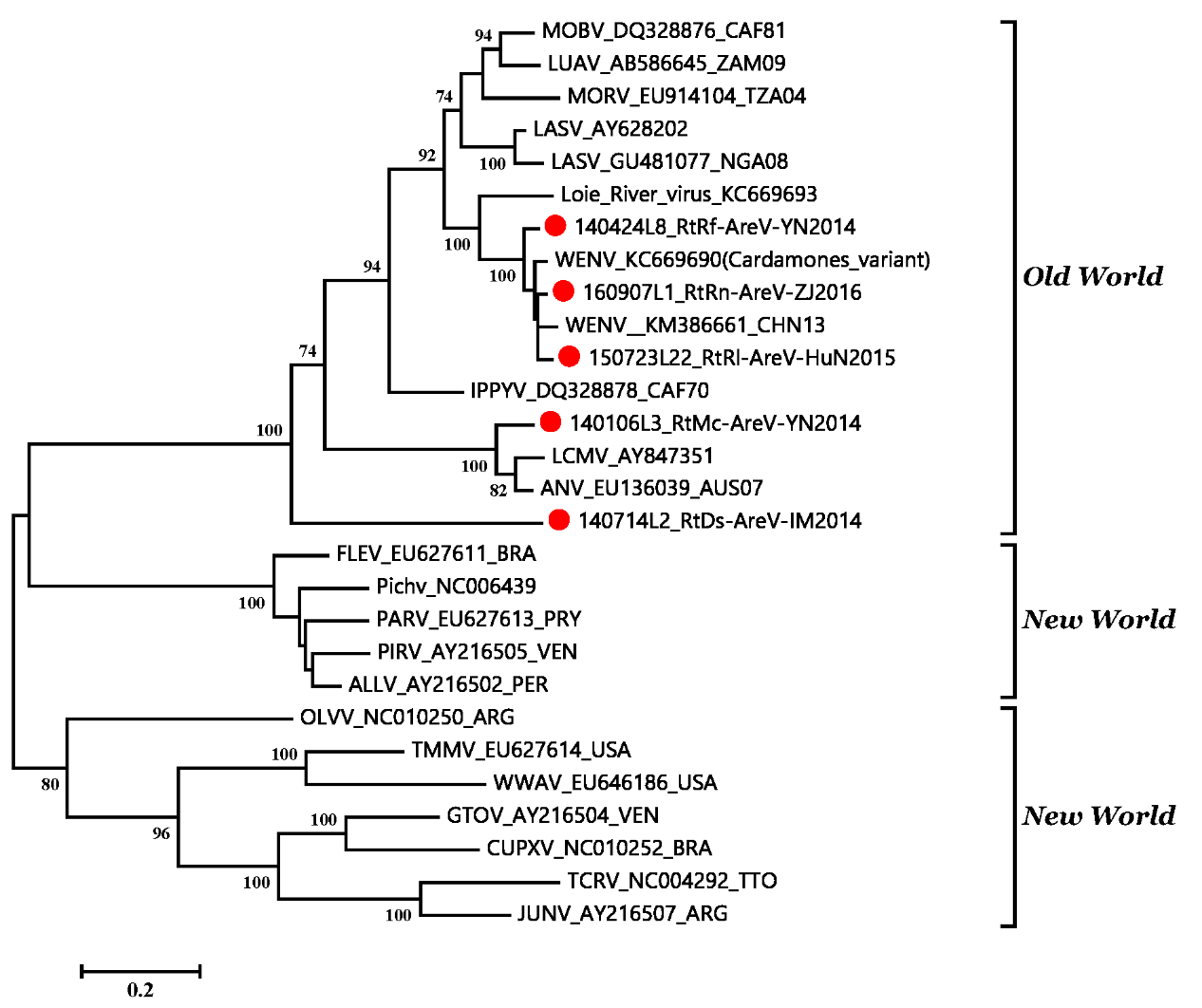


**Figure S1 Arenavirus G**. Phylogenetic tree showing the relationships (amino acid) between arenaviruses in the G polymerase. The viruses found in this study are labeled in red font.


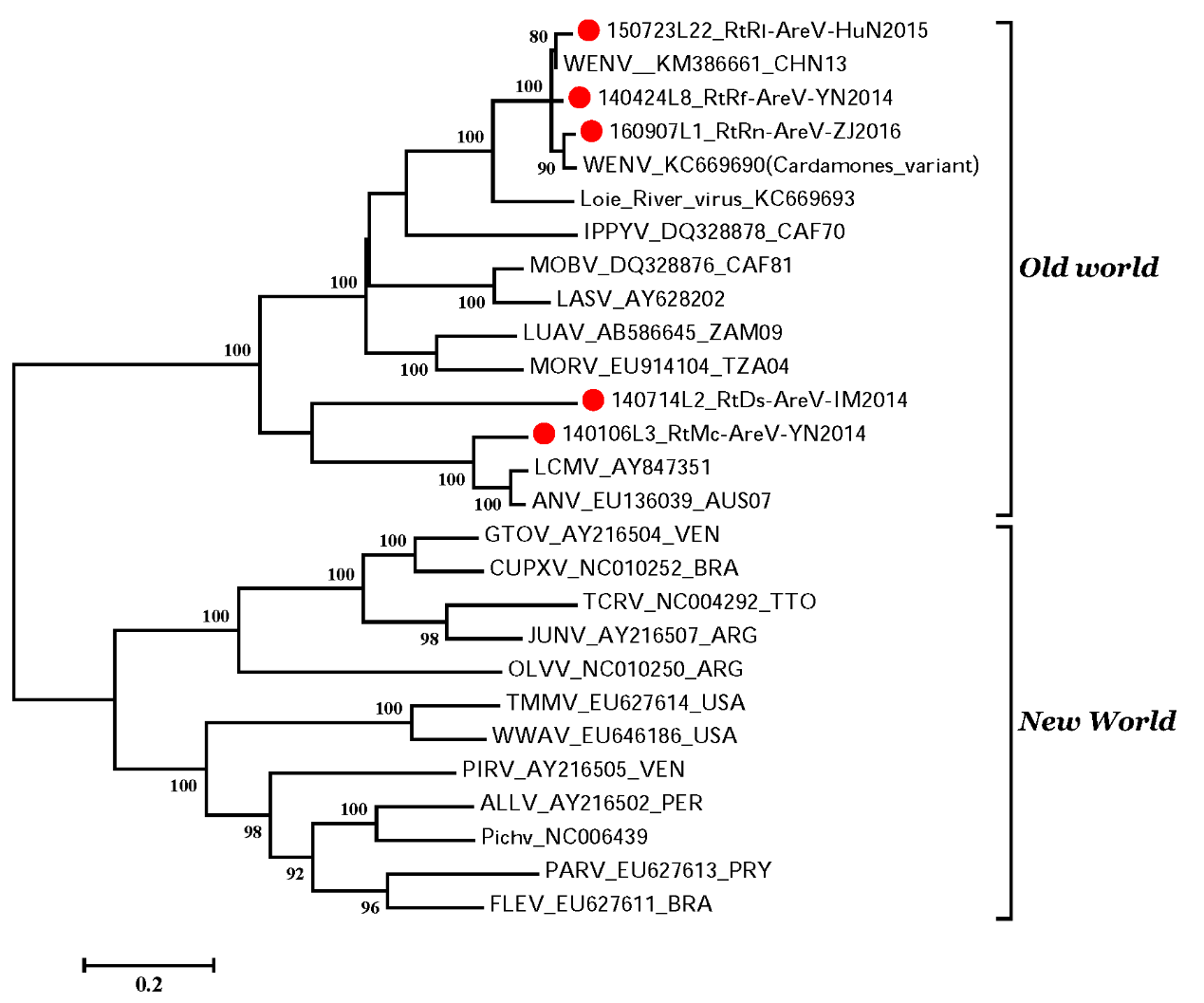


**Figure S2 Arenavirus N**. Phylogenetic tree showing the relationships (amino acid) between arenaviruses in the N polymerase. The viruses found in this study are labeled in red font.


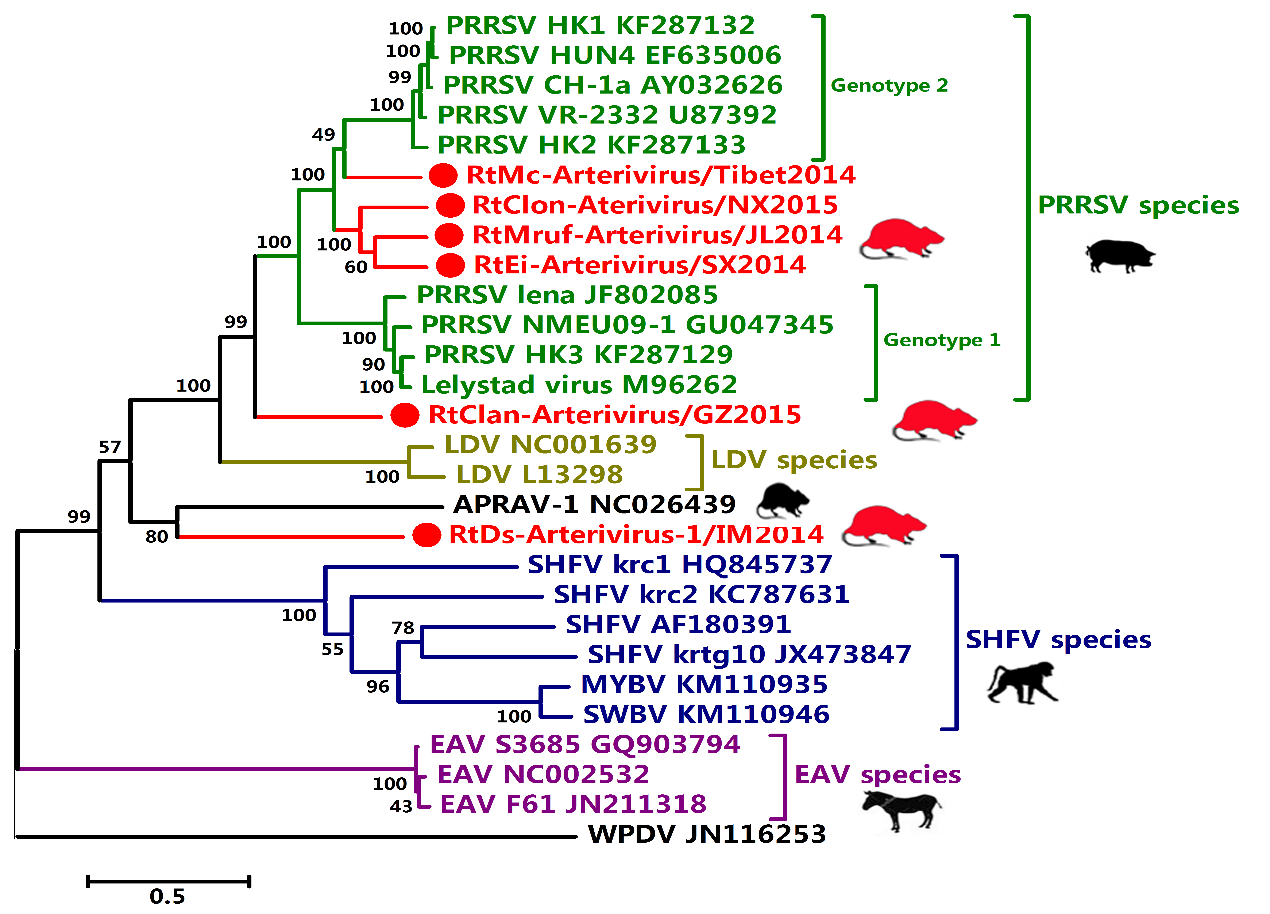


**Figure S3**. Phylogenetic tree showing the relationships (amino acid) between arteriviruses in the pp1a proteins. The viruses found in this study are labeled in red font.


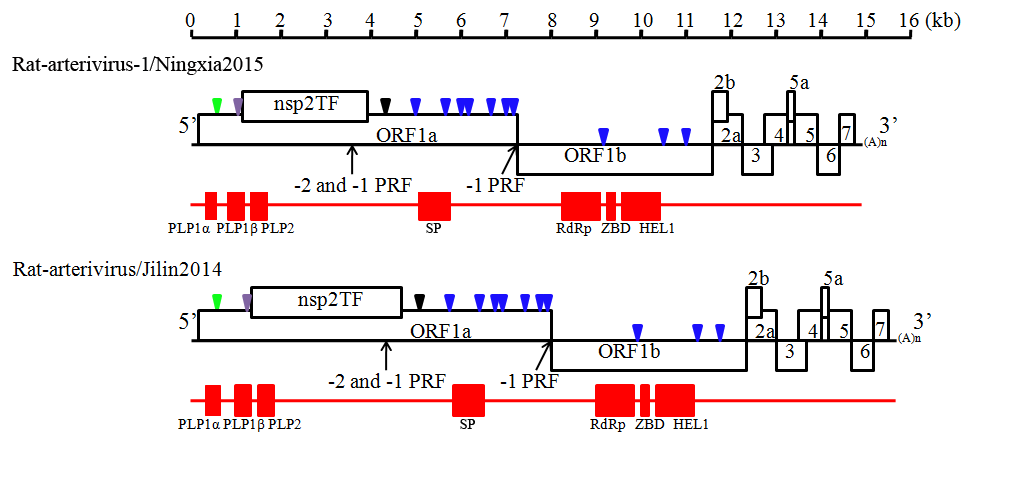


**Figure S4**. **Genomic organization of the Rat-arterivirus-1/Ningxia2015 and Rat-arterivirus/Jilin2014.** Black frame boxes represent the locations of the predicted ORFs and are drawn to scale. The PRF sites are marked with black arrows. The conserved domains of PLP1α, PLP1β, PLP2, SP, RdRp, ZBD, and HEL1 are marked with red boxes. The cleavage site of PLP1α is marked with a green triangle. The cleavage site of PLP1β is marked with a purple triangle. The cleavage site of PLP2 is marked with a black triangle. The cleavage sites of SP are marked with blue triangles.


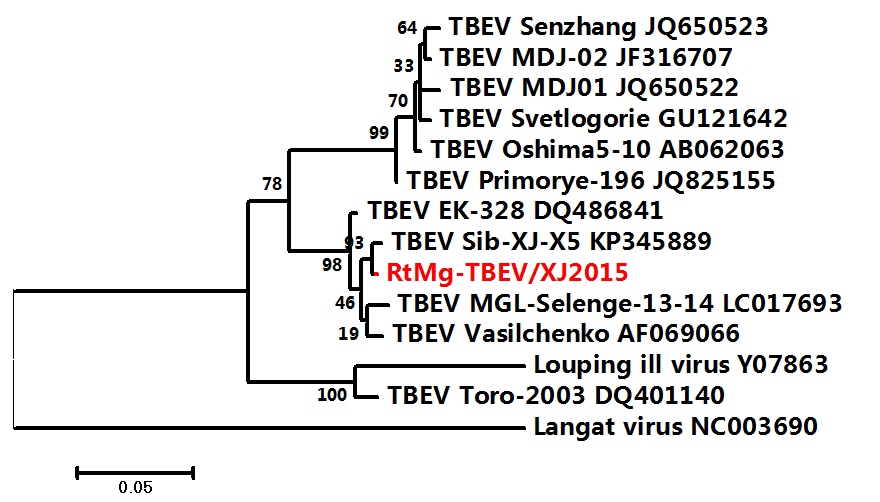


**Figure S5**. **phylogenetic tree based on the polyproteins of TBEV.** The viruses found in this study are labeled in red font.


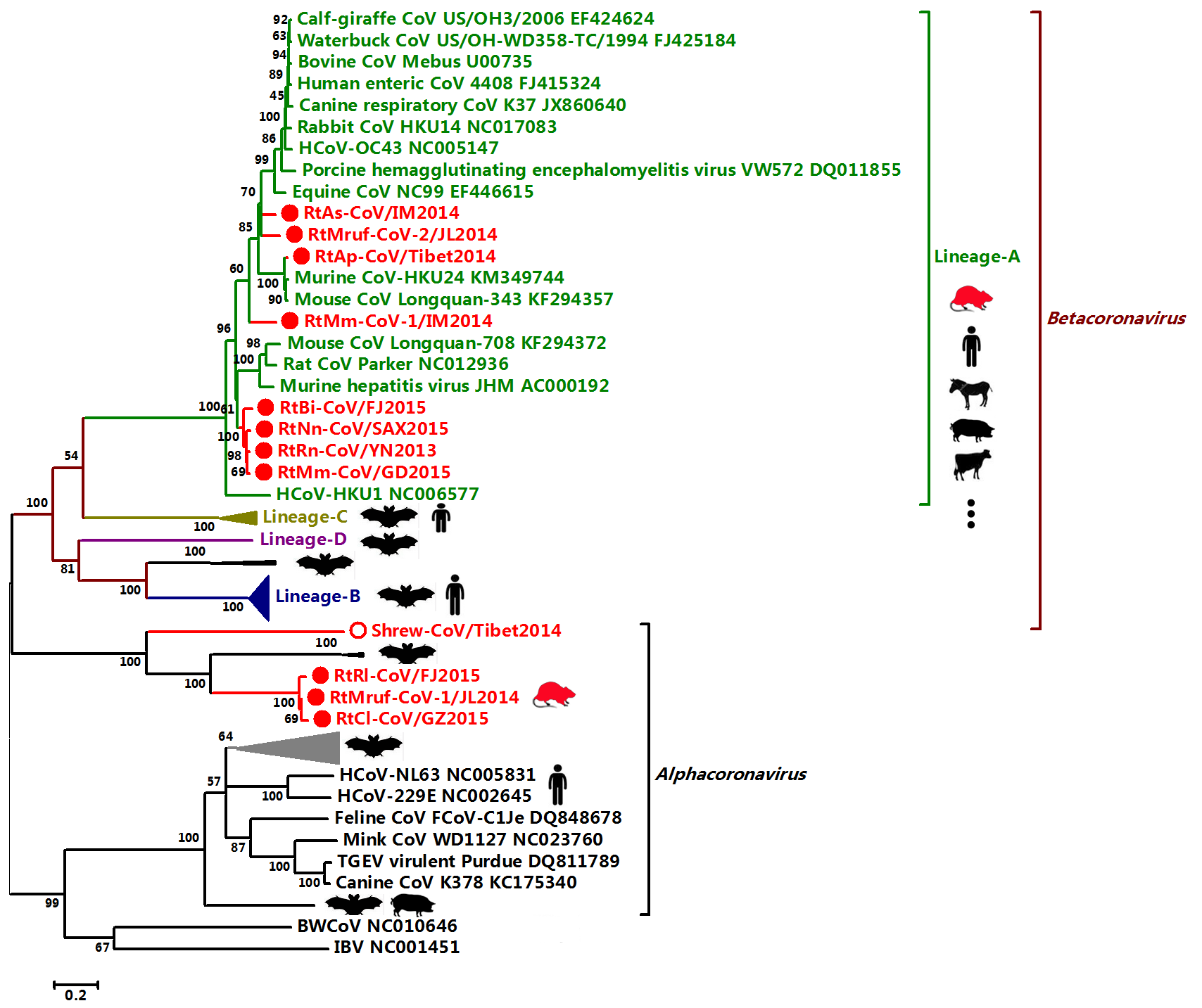


**Figure S6**. Phylogenetic tree based on the complete Spike (S) proteins of CoVs. The viruses found in this study are labeled in red font.


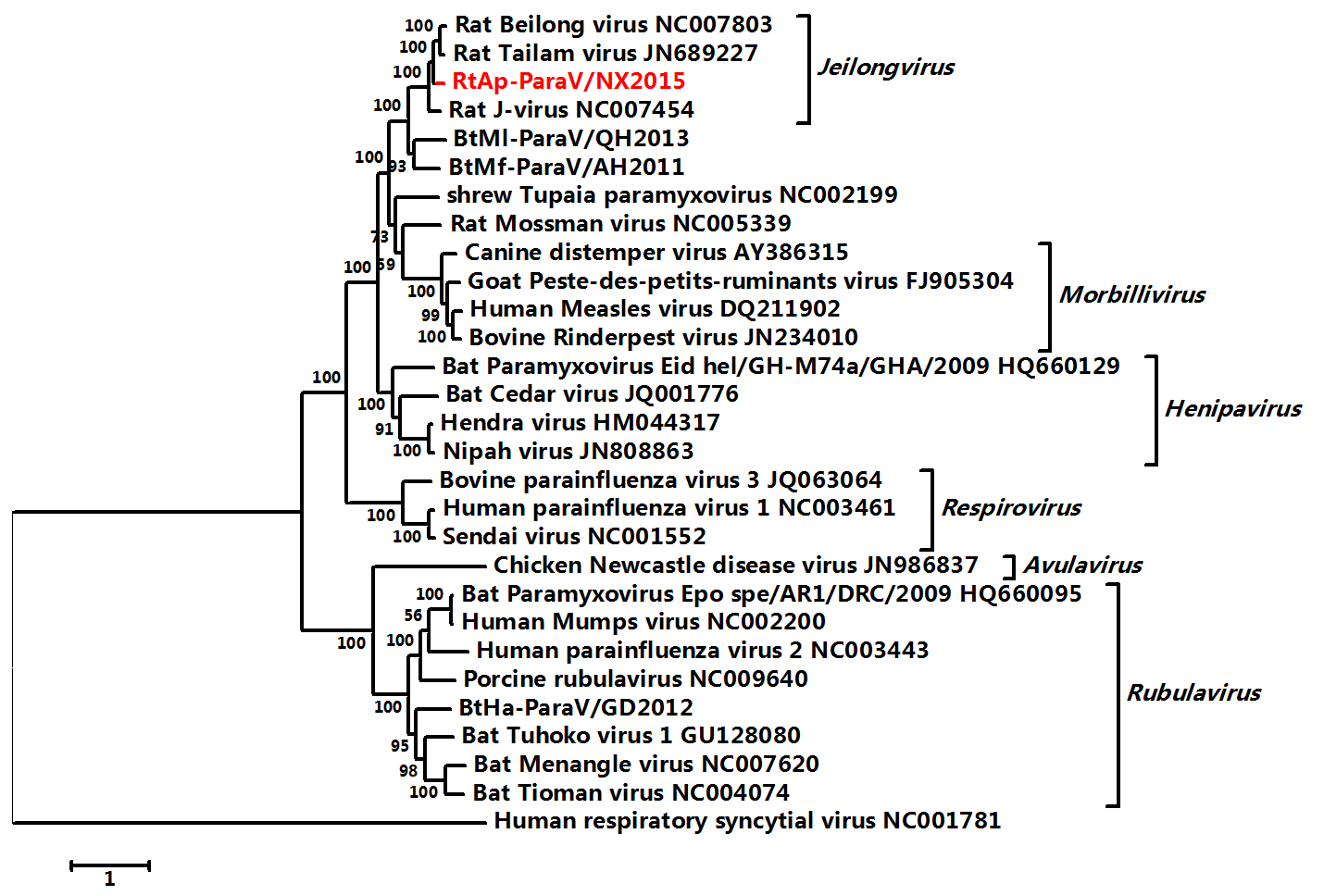


**Figure S7. Phylogenetic treebased on the L proteins of ParaVs.** The viruses found in this study are labeled in red font.


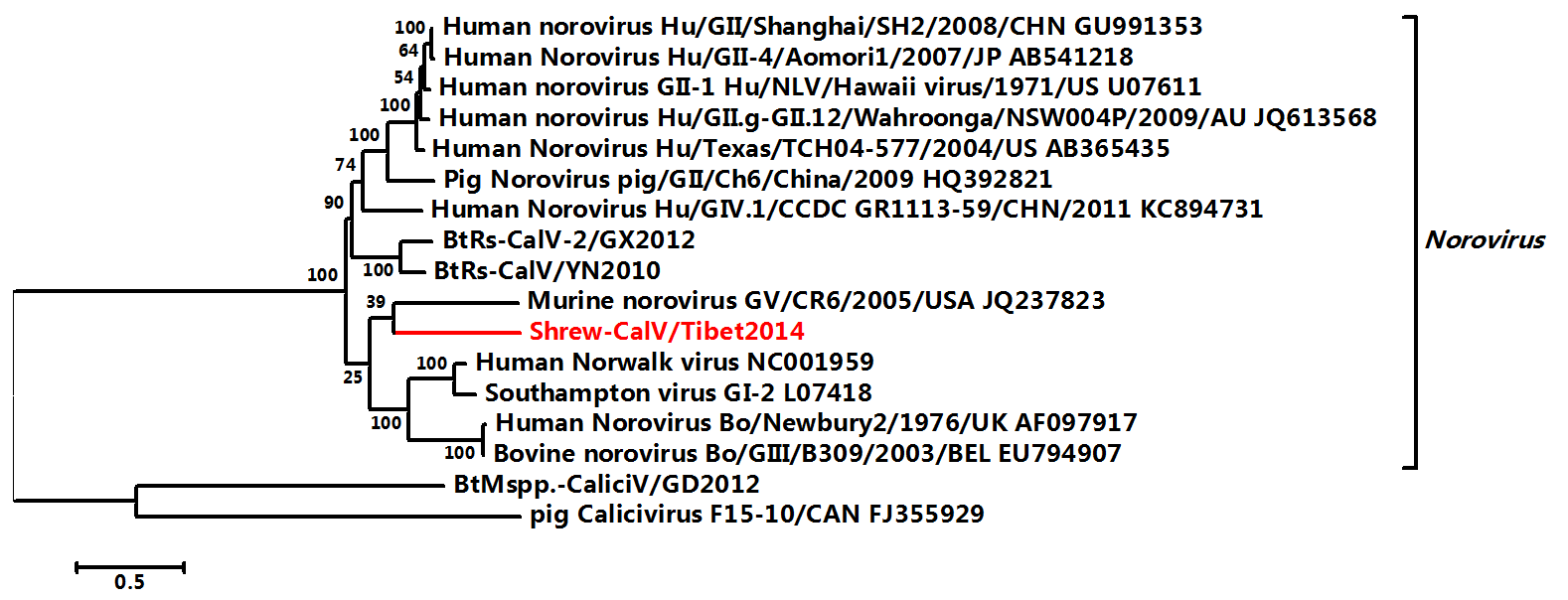


**Figure S8. Phylogenetic tree based on the polyproteins of Noroviruses.** The viruses found in this study are labeled in red font.


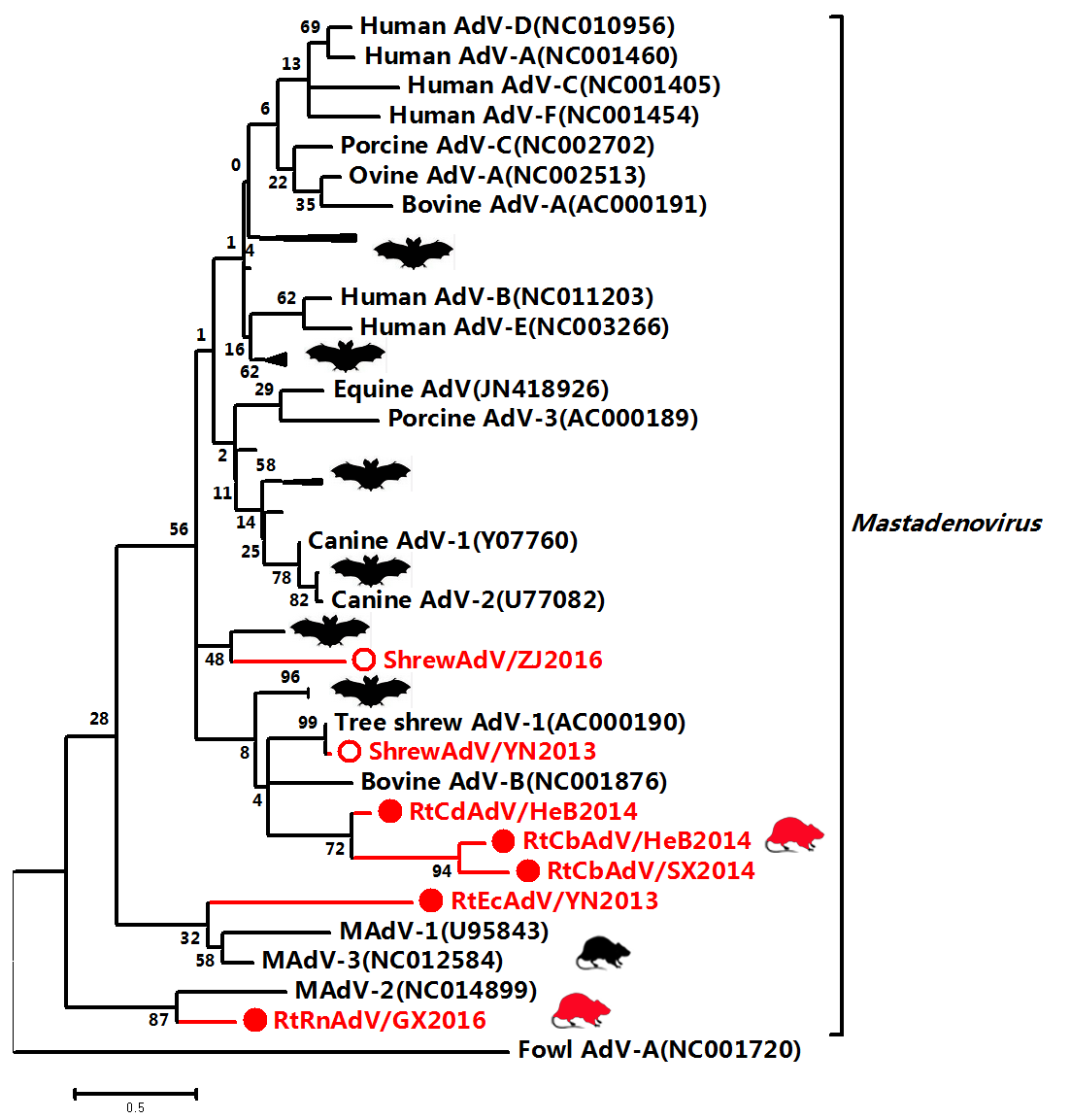


**Figure S9.** Phylogenetic tree based on diverse sequences of partial amino acid of the polymerases of AdVs. The viruses found in this study are labeled in red font.


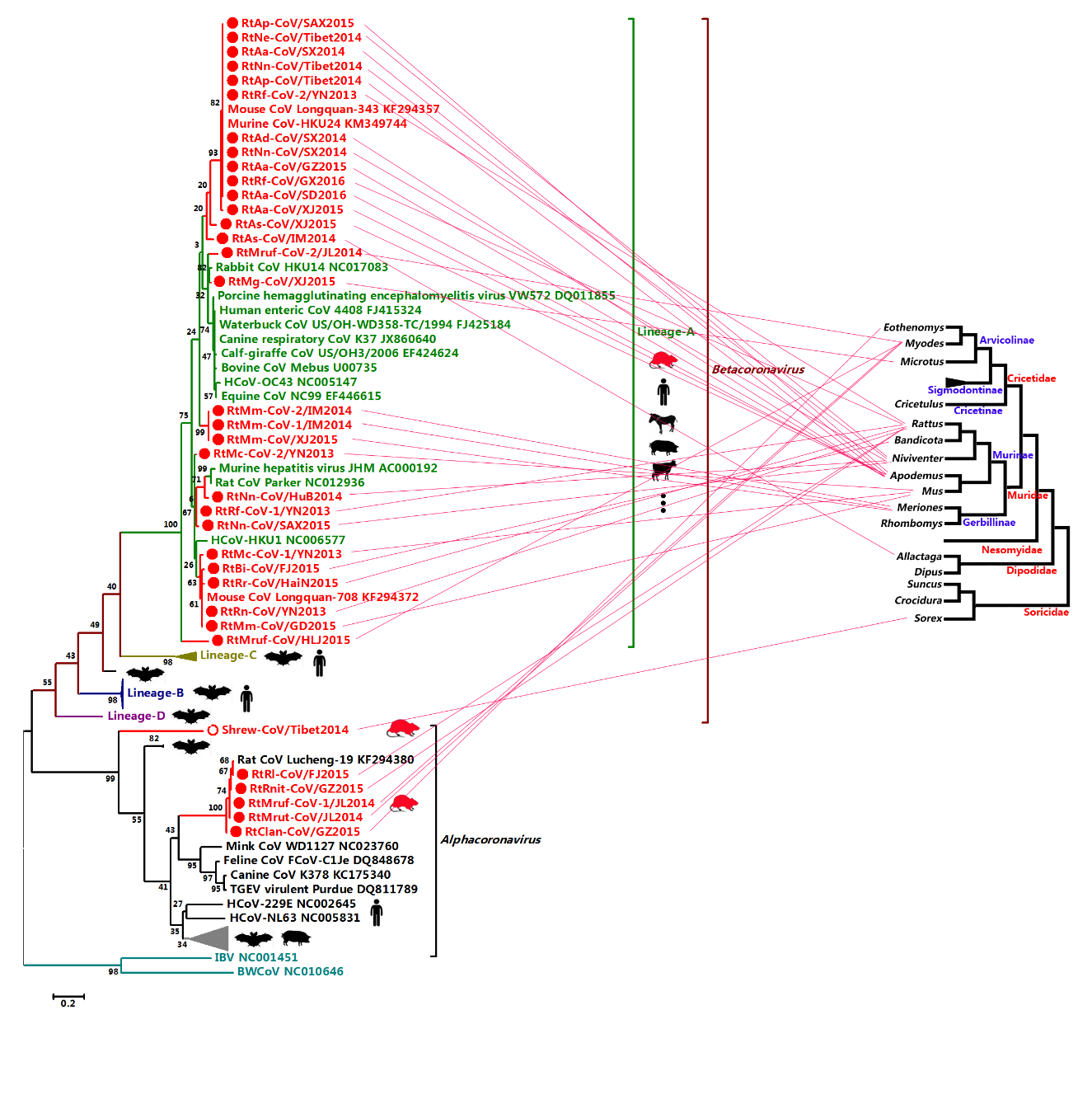


**Figure S10.** The phylogenetic relationships between CoVs and their hosts.


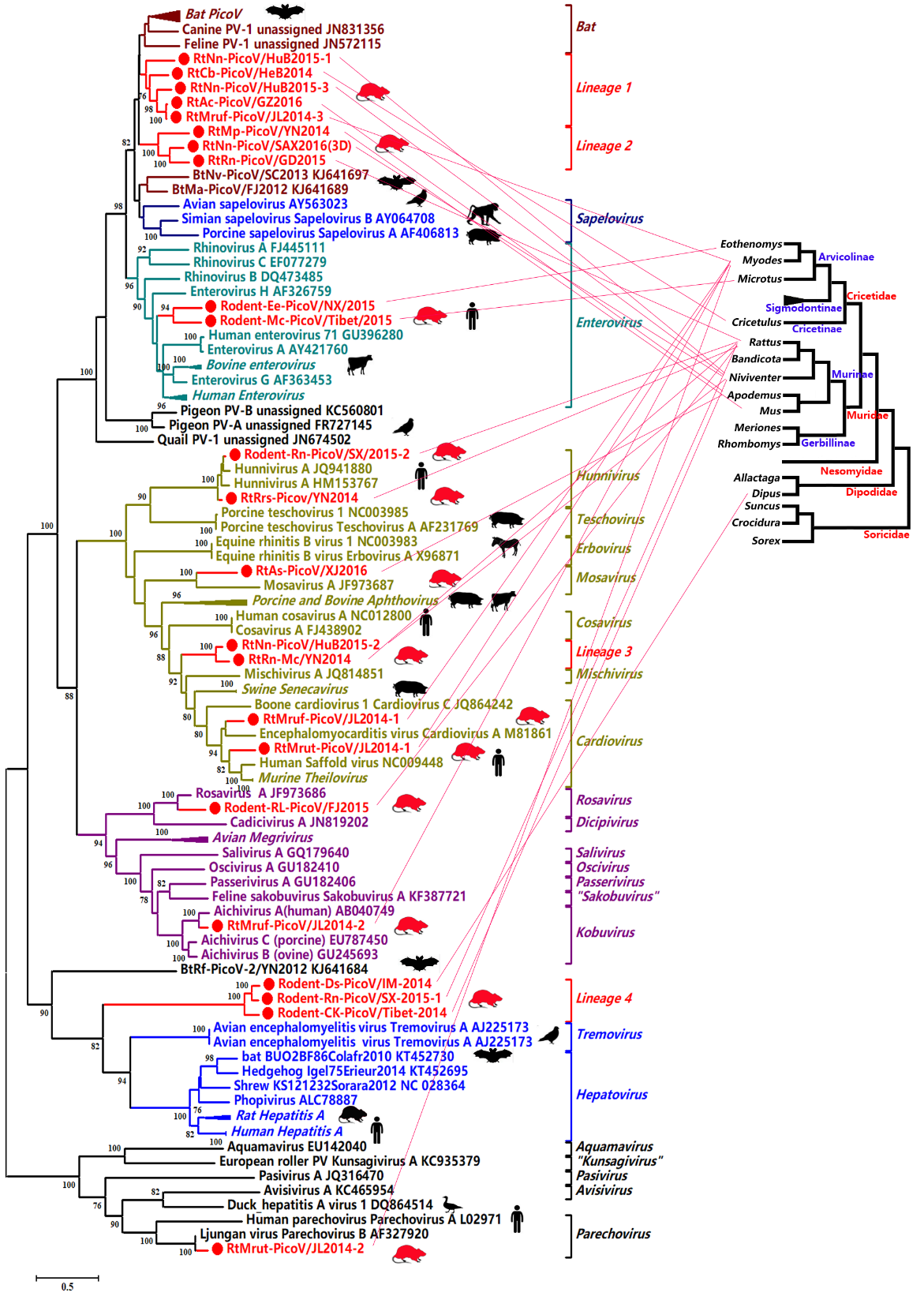


**Figure S11.** The phylogenetic relationships between PicoVs and their hosts.


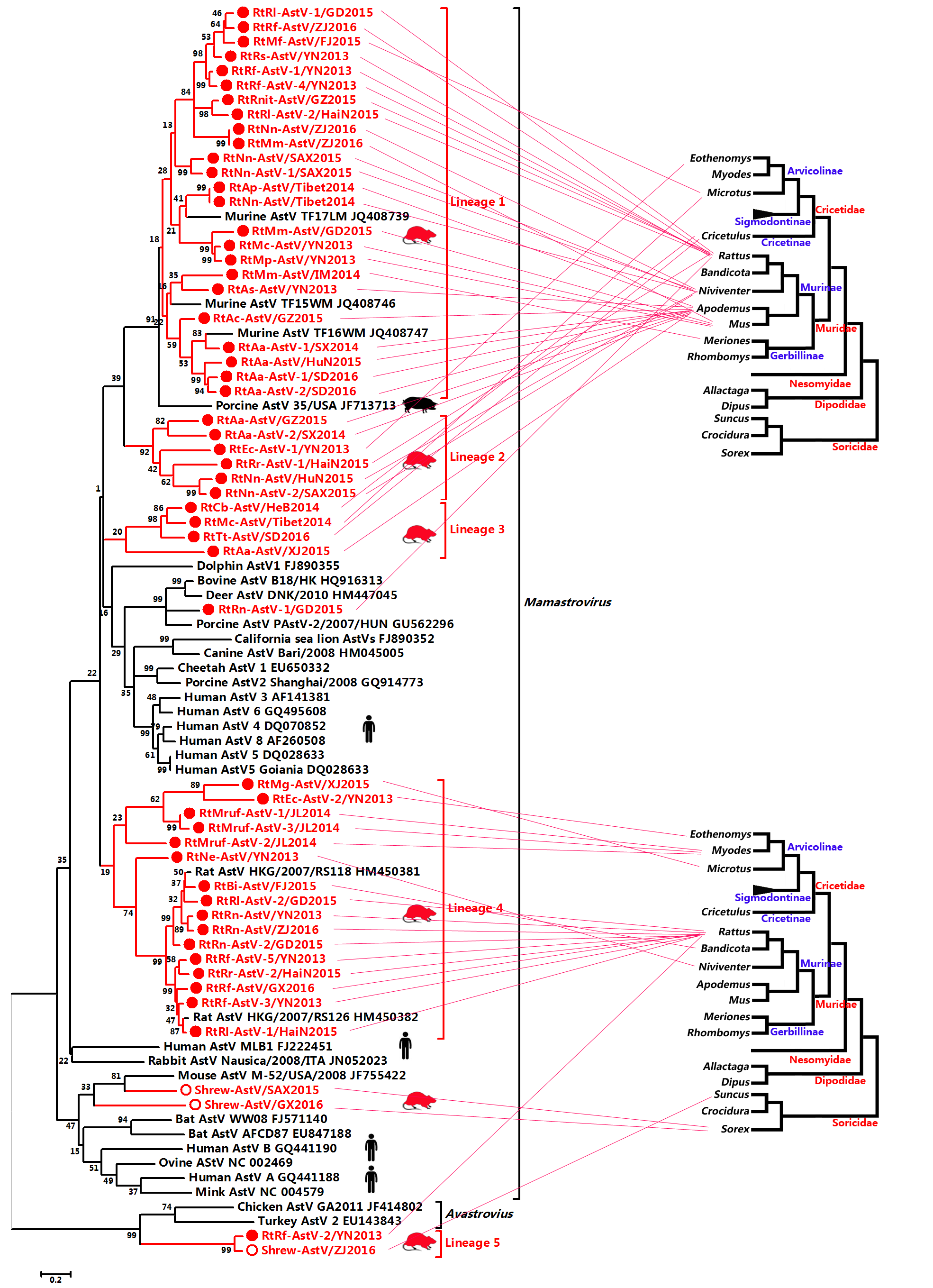


**Figure S12.** The phylogenetic relationships between AstVs and their hosts.


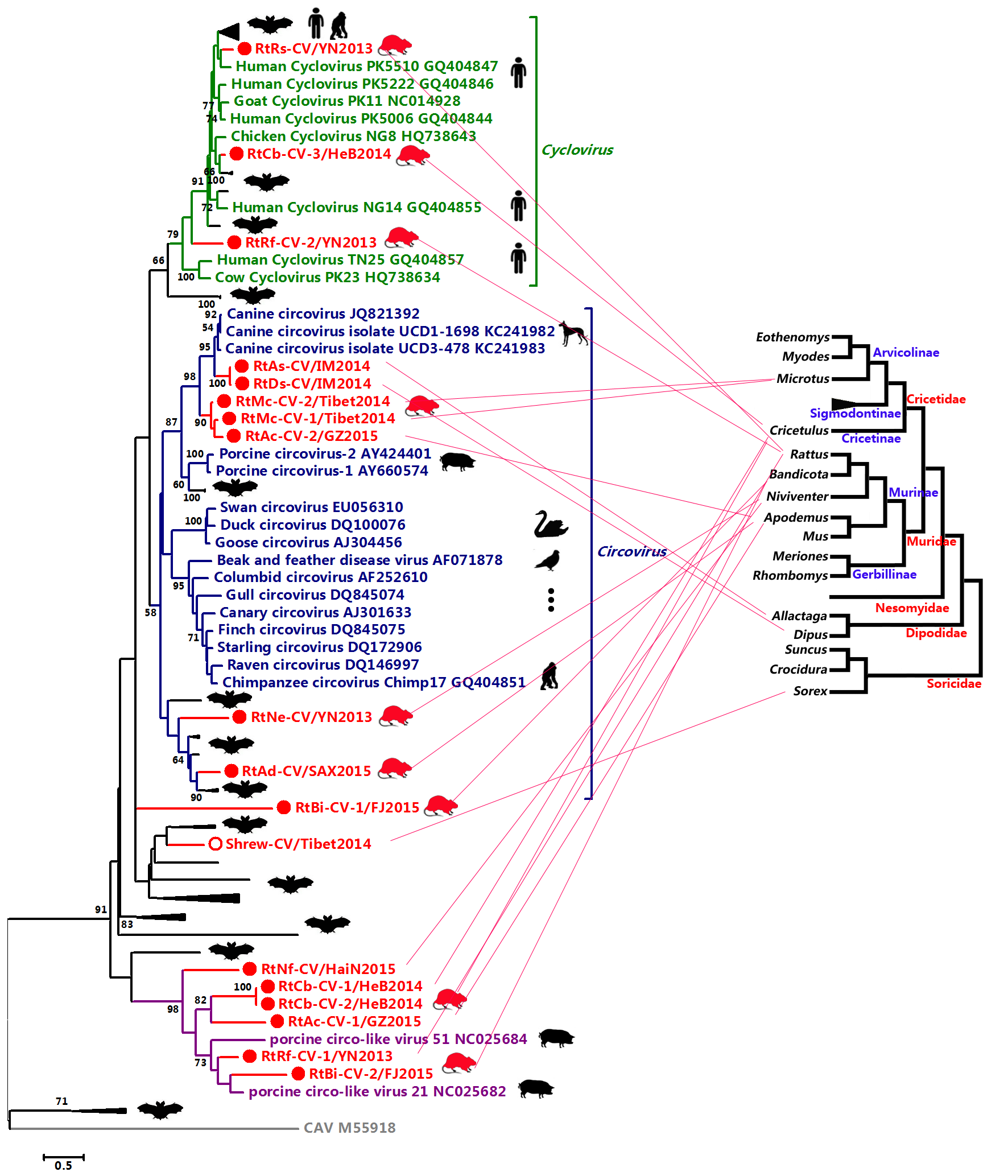


**Figure S13.** The phylogenetic relationships between CVs and their hosts.


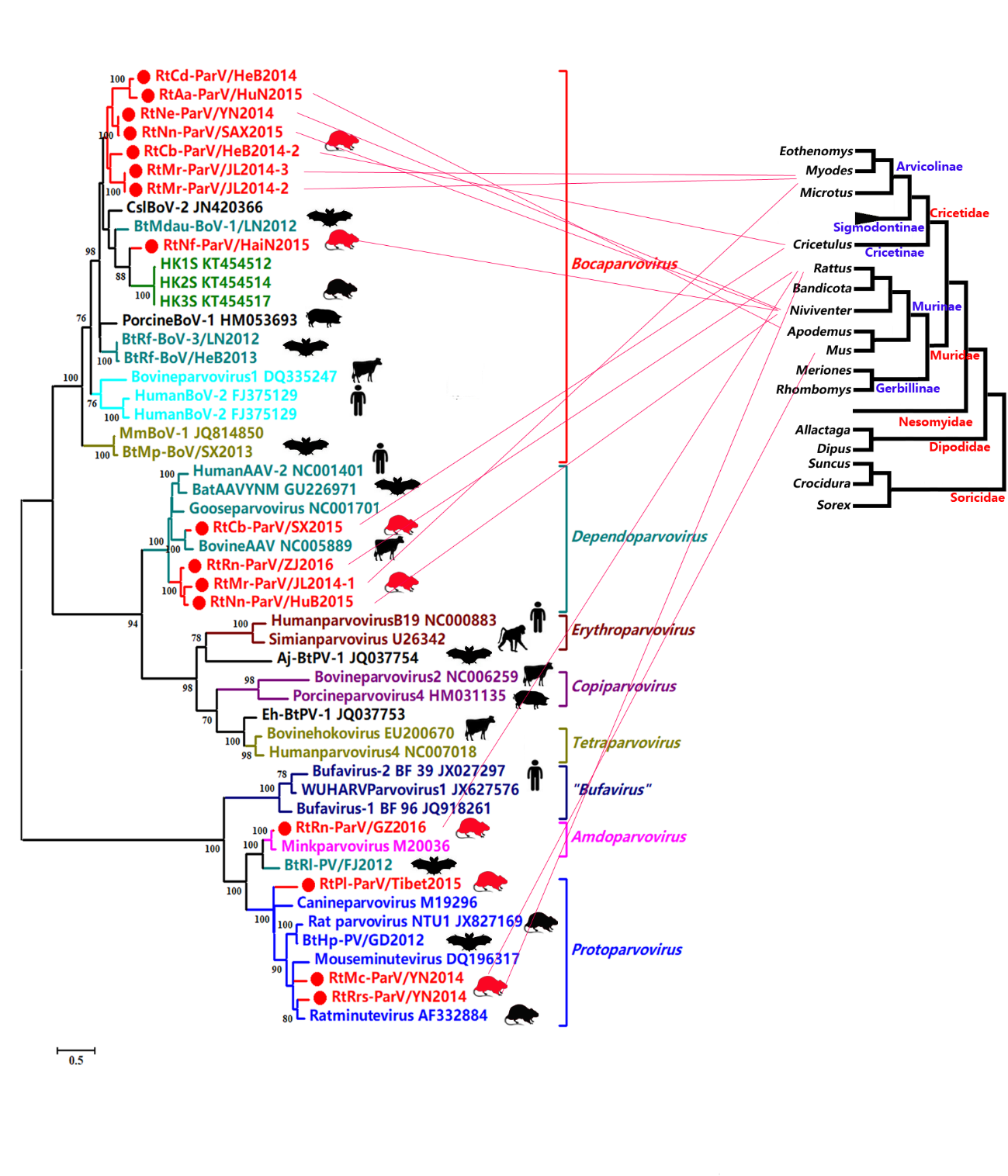


**Figure S14.** The phylogenetic relationships between ParVs and their hosts.
